# Deciphering the association between gene function and spatial gene-gene interactions in 3D human genome conformation

**DOI:** 10.1186/s12864-015-2093-0

**Published:** 2015-10-28

**Authors:** Renzhi Cao, Jianlin Cheng

**Affiliations:** Computer Science Department, University of Missouri, Columbia, Missouri 65211 USA; Informatics Institute, University of Missouri, Columbia, Missouri 65211 USA; Christopher S. Bond Life Science Center, University of Missouri, Columbia, Missouri 65211 USA

## Abstract

**Background:**

A number of factors have been investigated in the context of gene function prediction and analysis, such as sequence identity, gene expressions, and gene co-evolution. However, three-dimensional (3D) conformation of the genome has not been tapped to analyse gene function, probably largely due to lack of genome conformation data until recently.

**Methods:**

We construct the genome-wide spatial gene-gene interaction networks for three different human B-cells or cell lines from their chromosomal contact data generated by the Hi-C chromosome conformation capturing technique. The G-SESAME and Fast-SemSim are used to calculate function similarity between interacted / non-interacted genes. The Gene Ontology statistics computed from the gene-gene interaction networks is used for gene function prediction.

**Results:**

We compare the function similarity of gene pairs that do not spatially interact and that have interactions. We find that genes that have strong spatial interactions tend to have highly similar function in terms of biological process, molecular function and cellular component of the Gene Ontology. And even though the level of gene-gene interactions generally have no or weak correlation with either sequential genomic distance or sequence identity between genes, the interacted genes with high function similarity tend to have stronger interactions, somewhat shorter genomic distance and significantly higher sequence identity. And combining genomic distance or sequence identity with spatial gene-gene interaction information informs gene-gene function similarity much better than using either one of them alone, suggesting gene-gene interaction information is largely complementary with genomic distance and sequence identity in the context of gene function analysis. We develop and evaluate a new gene function prediction method based on gene-gene interacting networks, which can predict gene function well for a large number of human genes.

**Conclusions:**

In this work, we demonstrate that the spatial conformation of the human genome is relevant to gene function similarity and is useful for gene function prediction.

**Electronic supplementary material:**

The online version of this article (doi:10.1186/s12864-015-2093-0) contains supplementary material, which is available to authorized users.

## Background

As more and more genomes are sequenced, one urgent and important task in computational biology is to annotate and analyse the functions of the genes in a genome [1, 2]. A number of factors potentially related to gene function such as sequence identity, gene phylogenetic profiles, sequential genomic co-localizations, gene expressions, and protein-protein interaction have been investigated in the context of gene function prediction and analysis [3–8]. However, another very important aspect of a genome, i.e. three-dimensional (3D) conformation of the genome, which presumably plays an important role in organizing and regulating genes, has not been tapped to analyse gene function, probably largely due to lack of genome conformation data until recently.

Since the Hi-C technique [9] that can determine the genome-wide chromosomal interaction/contact data was invented in 2009, it has been applied to generate the large-scale genome-wide chromosomal conformation data for a number of genomes such as human B-cells [10, 11], yeast [12], bacteria [13], and Arabidopsis [14], which provides valuable data for studying the relationships between spatial gene-gene interactions and gene function. Similar technique has also been applied to study the three-dimensional model of budding yeast and other species [15, 16].

In this work, we analysed the intra- and inter-chromosomal interaction (contact) data of three different human malignant B-cell or cell lines (RL follicular lymphoma cell line (RL), primary tumor B-cells from an acute lymphoblastic leukaemia patient (ALL), and MHH-CALL-4 B-acute lymphoblastic leukaemia cell line (Call4)) [10] and one normal B-cell [9] captured by the Hi-C technique. From the Hi-C contact data, we generated the spatial gene-gene interactions for these cells or cell lines in order to investigate if the spatially interacting genes tend to have similar functions.

We compared the function similarity of spatially interacting gene pairs and non-interacting gene pairs in terms of three function categories of Gene Ontology [17]: Molecular Function (MF), Biological Process (BP) and Cellular Component (CC). Our analyses demonstrate that strongly interacting genes tend to have very similar function, and spatial gene-gene interaction is generally not or only weakly correlated with the sequential genomic distances between genes and with sequence identity between genes. However, strongly interacting genes with very similar function often have relative shorter average genomic distance and higher average sequence identity. Combining gene-gene interaction with either genomic distance or sequence identity can inform gene-gene function similarity better than either one of them. Furthermore, we developed a gene function prediction method based on spatial gene-gene interaction networks constructed from the Hi-C data. The method can rather accurately predict the function of a large number of genes based on their interaction with other genes, indicating the gene function prediction power of spatial gene-gene interaction information.

## Results

### The spatial gene-gene interaction network for whole genome and thresholds for substantially interacting gene pairs

We construct the gene-gene interaction network of the whole genome for the Hi-C data of three malignant B-cell/cell lines [10] and one normal B-cell [9]. A node and edge in the gene-gene interaction network represents the gene and spatial interaction between genes. In order to control the influence of the noisy chromosomal contacts in the Hi-C data, we consider that there existed a substantially interaction between two genes only if the number of chromosomal contacts observed between the two genes in the Hi-C data is greater than a pre-defined threshold. The interaction between two genes is considered strong when the number of contacts between them is greater than the pre-defined threshold. Higher the contact number, stronger is the interaction.

**Fig. 1:**
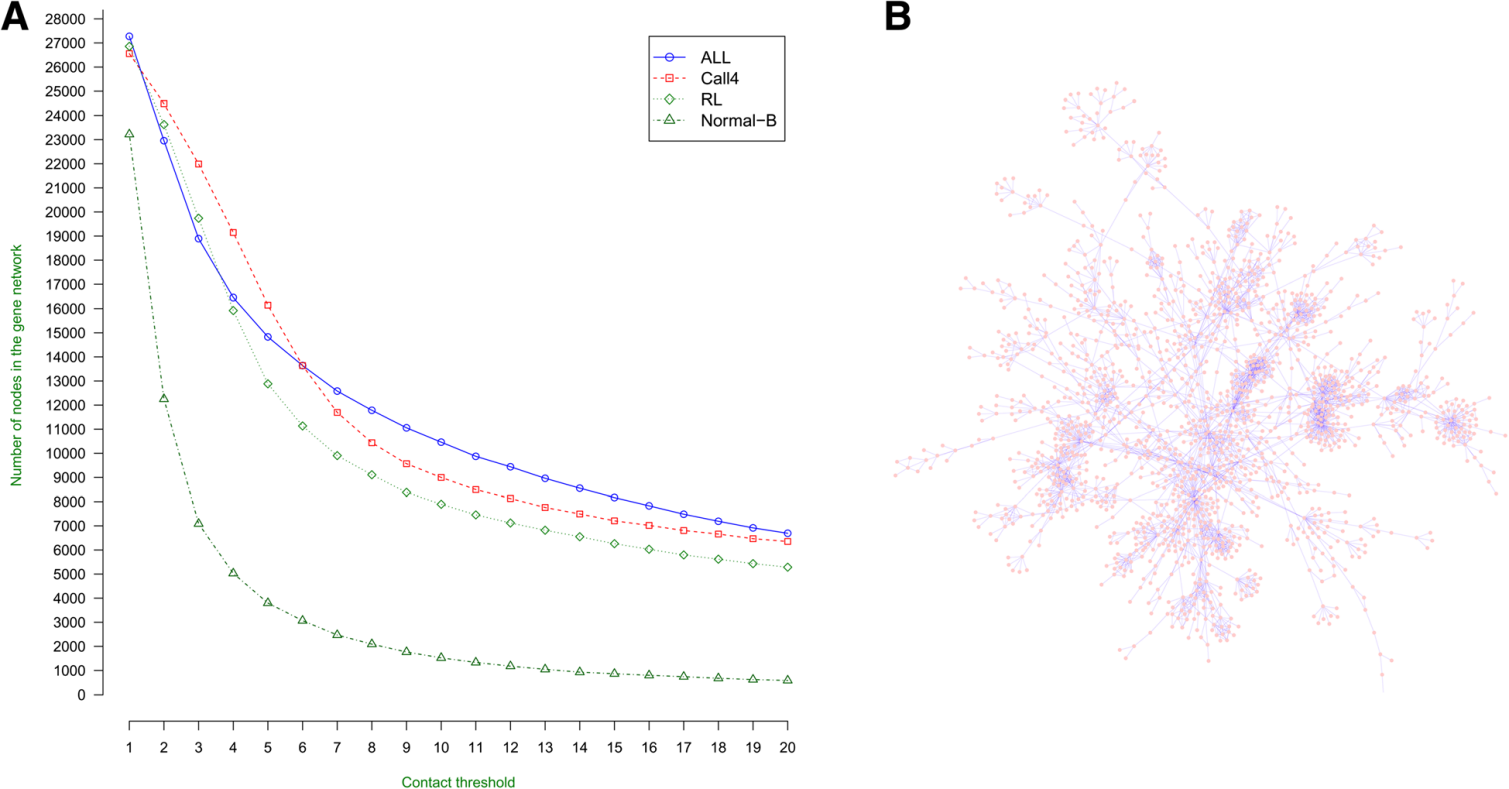
Visualization of gene-gene interaction network. Figure 1**a** is the plot of the numbers of interacted genes against interaction/contact thresholds for four cells/cell lines respectively. X-axis denotes the interaction thresholds and Y-axis the numbers of interacted genes found at the thresholds. Figure 1**b** is the visualization of the largest cluster of the gene-gene interaction network for the Call4 cell line at interaction threshold 16. The network was visualized by Cytoscape [24]

Since the number of chromosomal contacts automatically increases with respect to the total number of Hi-C reads in a Hi-C data, we set different thresholds on the four Hi-C datasets in order to make the number of the substantially interacting genes in these datasets largely the same. Actually, instead of using the number of nodes, similar threshold can be found on the four Hi-C datasets based on the number of edges in the interaction network. Figure 1a shows how the number of interacting genes in the spatial gene-gene interaction networks of the four Hi-C datasets changes with respect to the contact thresholds. The plot shows that the number of interacting genes / nodes decreases fast at the beginning and eventually levels off as the threshold increases. The decrease is most drastic on the spatial gene-gene interaction networks of the Normal B-Cell since the total Hi-C reads in its dataset is much smaller than the other three data sets. Assuming the number of interacting genes in the four interaction networks is similar, we set different thresholds on the datasets in order to select the same number of interacting genes in the Fig. 1a. Table 1 reports the thresholds used on each dataset in order to obtain ~7000 or ~12,000 interacting genes, respectively. These two sets of thresholds are selected because they are the only two thresholds that can lead to the similar number of interacted genes in the four cells/cell lines. About 7000 interacted genes can be found in all four cells / cell lines if the first threshold (the higher threshold) is used, and about 12,000 interacted genes are obtained if the second threshold (the lower one) is applied. According to Fig. 1a, the number of interacting genes changes relatively faster at around the second threshold than at around the first threshold. So, the first threshold leads to a more stable gene-gene interacting network, which is used for all the analysis in this work.

**Table 1:** Contact thresholds and the corresponding numbers of interacted genes for the spatial gene-gene interaction networks constructed for four cells/cell lines

	ALL	Call4	RL	Normal-B
Contact threshold	7	7	5	2
Number of gene nodes	12,581	11,693	12,882	12,251
Contact threshold	18	16	12	3
Number of gene nodes	7191	7019	7119	7089

Figure 1b illustrates the largest interacting gene cluster in the spatial gene-gene interaction network for the Call4 at the interaction threshold 16. At this threshold, 7019 genes were found to interact, which is close to the level-off point of the curves of the three malignant cells/cell-lines in Fig. 1a. All the genes that are connected by at least one path in the gene-gene interaction network are defined as a cluster. The cluster with largest number of genes is the largest cluster shown in the figure.

Additional file 1: Figure S1 shows the total number of nodes in the largest cluster with different interaction threshold for four different cell lines. As we can see from the figure, the total number of nodes in the largest cluster decreases rapidly at beginning, which shows a lot of edges in the network actually are formed with very few interactions. It is interesting to see that the total number of nodes in the largest cluster becomes stable with some interaction threshold for all four cell lines. As we use interaction threshold 12 for NORMAL-B cell, the number of nodes in the largest cluster is around 20, and it is stable even we increase the interaction threshold to 18. The 20 genes may play an important role in NORMAL-B cell. In addition, we use different interaction threshold for the other three cell lines (interaction threshold 204 for Call4, 157 for RL, 179 for ALL), so that the number of nodes in the largest cluster is also around 20, and the largest cluster is stable. We list these genes in Additional file 1: Table S1, the difference between the genes in NORMAL-B cell and other cell lines may help people to better understand these diseases.

### The function similarity of gene pairs that do not spatially interact and that have substantial interactions

**Fig. 2:**
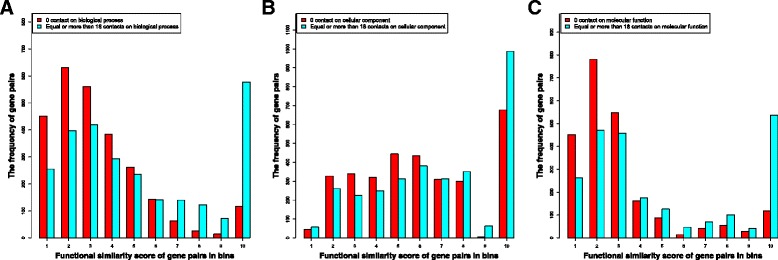
The histograms of gene function similarities of non-interacted gene pairs and substantially interacted gene pairs. Figure 2**a**, **b**, and **c** represent the histogram for Biological Process, Cellular Component, and Molecular Function respectively

We compare the function similarity of gene pairs that substantially interacted (i.e., Hi-C contact number > = a predefined threshold) and that did not interact in terms of Gene Ontology (GO) function definitions. Figure 2 shows the histogram of the function similarity of non-interacting gene pairs and interacting gene pairs in the three GO categories (BP, CC, MF), respectively. The interacting gene pairs were selected from the genes that had > = 18 Hi-C contacts and the non-interacted pairs were the ones randomly selected that had no Hi-C contacts according to the Hi-C data of the ALL cell. The most obvious difference in the function distribution is that substantially more interacting genes had almost identical function (i.e. similarity bin 10 in the figure) than the non-interacting genes, while fewer interacting gene pairs fell into other function similarity bins than non-interacting gene pairs. This is the case for all three GO function categories, even though the level of the difference in the function similarity bin 10 is somewhat different. In order to identify the interacting genes with highly similar functions, we calculate the statistics of the number of spatial interactions for the gene falling into different function similarity bins.

### The statistics of the number of interactions for substantially interacting gene pairs at each function similarity level

**Fig. 3:**
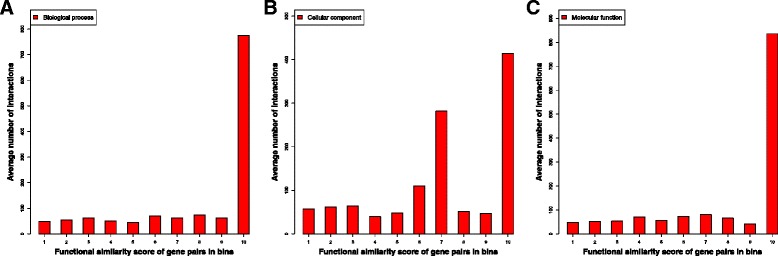
The average number of interactions between substantially interacted gene pairs within each functional similarity bin in three function categories. This is for the primary tumor B-cells (ALL). Figure 3**a**, **b**, and **c** represent the histogram for Biological Process, Cellular Component, and Molecular Function respectively

Figure 3 shows the average number of observed chromosomal interactions for the gene pairs in each function similarity bin in each GO function category. It is very interesting to see that the average number of interactions between genes in function similarity Bins 1–9 is rather similar, while the average number of interactions for the genes in Bin 10 is much higher. The average numbers of interactions between genes in function similarity bins 9 and 10 for three function categories (BP, CC, MF) are (62.22, 775.12), (46.54, 414.28), and (41.61, 835.80), respectively. According to the Welch two-sample *t*-test, the p-value of the difference in the average numbers of interactions between bin 9 and bin 10 is less than 2.2e-16 for all three categories. This indicates that the interacting genes with almost identical functions are more strongly interacted than the rest of interacting gene pairs. In the other words, the strongly interacting genes tend to have almost identical function. Similar pattern is found in other cell lines, which is illustrated in Additional file 1: Figure S2.

Since a few outliers (extremely large numbers) may skew the average number substantially, we also calculated the quantiles of the interaction numbers in the function similarity bins (see Additional file 1: Figure S3). Indeed, the genes in function similarity Bin 10 have substantially more interactions than genes in the other bins. For example, the median interaction number and the quantile at 75 % in Bin 10 for Biological Process is 407 and 1187, which are much higher than 31.5 and 47.75 in Bin 9. Interestingly, the genes in the other bins except Bin 10 seem to have similar median interaction numbers despite their different levels of function similarity.

### The sequential genomic distance for substantial-ly interacting gene pairs at each function similarity level

**Fig. 4:**
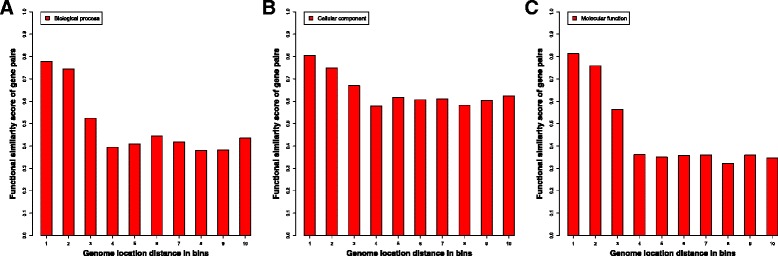
The average genomic distances of substantially interacted gene pairs in each functional similarity bin in three function categories. This is for the primary tumor B-cells (ALL). Figure 4**a**, **b**, and **c** represent the histogram for Biological Process, Cellular Component, and Molecular Function respectively

We gauge the relationship between the sequential genomic distances of interacting gene pairs in function similarities. Figure 4a, b and c illustrates the average function similarity in each genomic location distance bin for Biological Process, Cellular Component and Molecular Function, respectively. Gene pairs are classified into ten bins based on their genomic location distance, and each bin has the same number of gene pairs. The gene pairs are substantially interacting genes (> = 18 Hi-C interactions) identified in the Hi-C data of the ALL cell. The genomic distance between two genes is the number of base pairs between their start locations. Since it is difficult to define the sequential genomic distance between genes on two different chromosomes, inter-chromosomal gene pairs were not considered in the calculation. The results show that gene pairs with short genomic distances usually have high function similarity. For example, gene pairs in the first three bins have high function similarity comparing with gene pairs in other bins for all three categories. Especially for Biological Process and Molecular Function, the function similarity of Bin 1 (relatively in short genomic distance) is around two times higher than the function similarity of Bin 10.

In order to reduce the influence of some genes with extremely large genomic distance, we generated the box plots for genomic distances in each function similarity bin for each function category (see Additional file 1: Figure S4). The result shows that the median genomic distance of all gene pairs with functional similarity score (<0.9 in Bins 1–9) is longer than the ones with very high functional similarity score (>0.9 in Bin 10). For example, for biological process category, the median genomic distance in Bin 1 is 574,281 bp, longer than 72,312 bp in Bin 10; for the cellular component, the median genomic distance in Bin 1 is 458,991 bp, longer than 201,949 bp in Bin 10; and for the molecular function, the median genomic distance in Bin 1 is 565,609 bp, longer than 64,167.5 bp in Bin 10. In summary, the genomic distance can somewhat distinguish the interacting gene pairs with very high function similarity from the rest of interacted pairs. However, its effect is more pronounced on Biological Processes and Molecular Function than on Cellular Component.

Similarly, we calculated the genomic distances for 20,000 randomly selected gene pairs in ten function similarity bins that did not spatially interact (see the boxplots in Additional file 1: Figure S5). In contrast to the interacting gene pairs, the median genomic distances are relatively close for non-interacting gene pairs in different bins, and gene pairs in high function similarity bins do not always have minimum median genomic distances. Furthermore, the genomic distance of gene pairs with no interaction is relatively longer than substantially interacting gene pairs in different functional similarity bins.

### Sequence identity of substantially interacting genes at each function similarity level

**Fig. 5:**
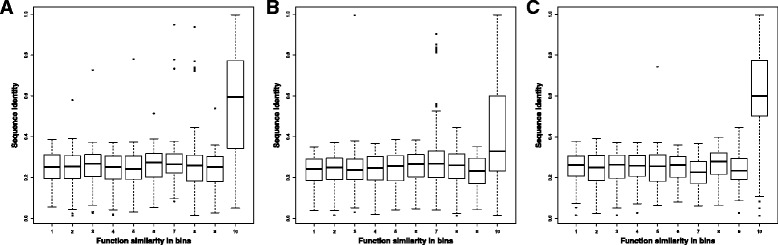
The boxplot of gene sequence identity against function similarity in three GO categories. Figure 5**a**, **b**, and **c** represent the histogram for Biological Process, Cellular Component, and Molecular Function respectively. This figure is generated on the gene-gene interaction network of the ALL B-cell constructed at interaction threshold 18. X-axis denotes the functional similarity scores / bins and Y-axis gene sequence identity

We assessed the relationship between sequence identity and function similarity for substantially interacting gene pairs (> = 18 Hi-C contacts) in the Hi-C data of the ALL cell line. Figure 5a, b and c illustrates the box plots of the sequence identity of gene pairs in 10 function similarity bins for Biological Process, Cellular Component, and Molecular Function, respectively. The median sequence identity of gene pairs in Bin 10 (i.e. similarity score in [0.9, 1]) is generally higher than the rest bins, even though the difference is more pronounced for Biological Process and Molecular Function than Cellular Component. For Biological Process and Molecular Function, the median sequence identity in Bin 10 is about 0.6, and for Cellular Component, the median sequence identity of gene pairs in Bin 10 is about 0.4. The median sequence identity in other 9 bins for each function category is similar to each other and substantially lower than Bin 10, even though there are quite some outliers in Bin 10 that have very low sequence identity. The histogram of the average sequence identity for substantially interacting gene pairs in each functional similarity bin is reported in Additional file 1: Figure S6. Moreover, the sequence identity calculated by Needle-Wunsch algorithm is also included in the figure to make comparison with the one by dynamic programming technique. This figure shows that the average sequence identity in Bin 10 is much higher than most other bins for each category. Interestingly, the average sequence identity increases as the function similarity bin increases, and the average sequence identity in Bin 10 for each category is always relatively high. Therefore, the sequence identity could be a factor to predict if two interacting genes have very high functional similarity score (> = 0.9). The substantially high sequence similarity between interacting genes with high function similarity may be partially due to the duplicated genes that still maintain highly similar functions and are spatially close [18, 19].

### Identification of interacting genes with high function similarity with sequence identity, genomic distance, and interaction strength

Since the special group of interacting genes with function similarity score > = 0.9 tend to have higher sequence identity, shorter genomic distance, and stronger spatial interactions, we tested how these three factors could identify this group of genes. Additional file 1: Figure S7 reports the number of gene pairs with functional similarity score > = 0.9 identified by setting on thresholds on the interaction number, sequence identity, and genomic distance of substantially interacting genes (> = 18 Hi-C contacts) in the Hi-C data of the ALL B-cell, for Biological Process (Additional file 1: Figure S7 (A)), Cellular Component (Additional file 1: Figure S7 (B)), and Molecular Function (Additional file 1: Figure S7 (C)), respectively. The threshold on interaction numbers is set to 50, genomic distance to 1,000,000 bp for Biological Process and Molecular Function and 2,000,000 bp for Cellular Component, and sequence identity to 25 %.

The results shows that applying the thresholds on the three factors can identify 372–398 common interacting gene pairs with high function similarity for each function category, while using each threshold can identify some gene pairs not recognized by another factor. Applying sequence identity or genomic distance to interacting genes can identify more gene pairs with high function similarity than using interaction number, suggesting combining sequence identity or genomic distance with gene spatial interaction information could be more sensitive in identifying genes with high function similarity than using interaction information alone. In general, the substantial number of common gene pairs identified by each of the three factors demonstrates the convergence in the group of interacting genes with high function similarity and the distinct gene pairs found by each factor also suggests the complementarity of the three factors.

### The relationship between sequence identity and function similarity for substantially interacting gene pairs and random non-interacting gene pairs

**Fig. 6:**
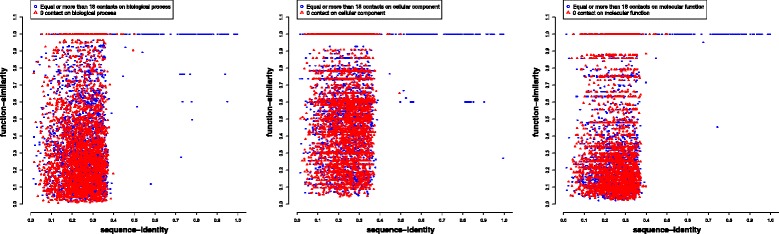
Plot of function similarity against sequence identify for substantially interacted gene pairs and non-interacted gene pairs. X-axis denotes the gene sequence identity and Y-axis the gene function similarity in all three categories (BP, CC, MF), respectively. The blue circle reprensents gene pairs with equal or more than 18 contacts in each categories (BP, CC, MF), and the red triangle is for gene pairs with no contacts in each categories

Figure 6 plots function similarity against sequence identity of 7987 interacting genes pairs with > = 18 Hi-C contacts (excluding ones without GO annotations) and 20,000 randomly selected, non-interacting gene pairs in the gene-gene interaction network of the ALL cell line. For non-interacting gene pairs, the correlation between sequence identity and function similarity is very low, i.e., 0.02, 0.05, and 0.03 in three function categories (i.e. BP, CC, and MF). In contrast, for the substantially interacting gene pairs, the correlation score is much higher, i.e., 0.67, 0.41, and 0.70 for three function categories, respectively. In order to compare the function similarities of interacting genes and non-interacting genes more rigorously, we also select non-interacting gene pairs by restricting their genomic distances are similar to the selected highly interacting gene pairs (within 35 bp). Additional file 1: Figure S8, S9, S10, S11 plot the function similarity against sequence identity for highly interacting gene pairs and random gene pairs with similar genomic distance for four cell/cell lines. The correlation between sequence identity and function similarity for non-interacting random gene pairs with the genomic distance restriction is higher than that of non-interacting random gene pairs without the genomic distance restriction, but is still lower than that of substantially interacting gene pairs. For example, the correlation between sequence identity and function similarity for these three gene groups in the ALL cell is 0.37, 0.25, and 0.43 respectively. This suggest both genomic distance and spatial gene-gene interaction between gene pairs affect the correlation between their sequence identity and function similarity, and spatial gene-gene interaction further strengthens the correlation when the genomic distance between genes is similar.

Additional file 1: Figures S12 and S13 visualize how function similarity changes with respect to sequence identity for non-interacting gene pairs and substantially interacting gene pairs. The results show that there is a much stronger correlation between sequence identity and function similarity for substantially interacting gene pairs than non-interacting gene pairs.

**Fig. 7:**
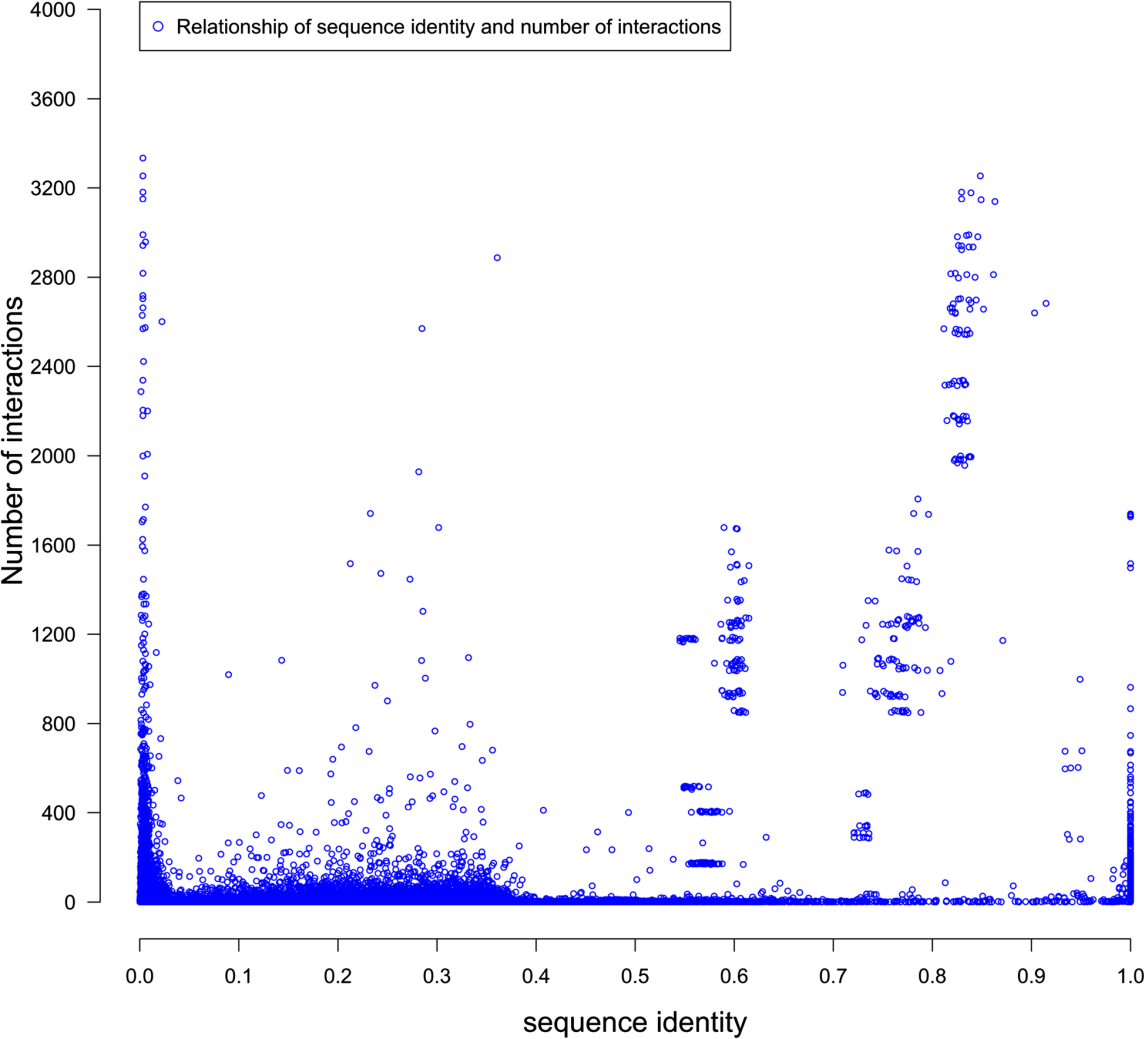
The sequence identity and the number of gene interactions. The number of interactions is normalized to the range of 0 to 1. The result is generated on the ALL gene-gene network with > =1 interactions. X-axis denotes the sequence identity and Y-axis the normalized number of interactions

Figure 7 plots the numbers of interactions of gene pairs against their sequence identities. The top 20 points with extremely large number of interactions are removed. According to the plot, the number of interactions varies a lot when sequence identity is either around 0 or 1. Indeed, the Pearson’s correlation between sequence identity and the number of interactions for all spatially interacting gene pairs is only 0.223. Additional file 1: Figure S14 shows the number of interaction of gene pairs against their sequence identities for the other three cell lines. Similar pattern has been discovered.

The weak correlation between interaction numbers and sequence identity and the relatively strong function prediction power of considering both sequence identity and interaction numbers suggest that they are two rather independent factors informing the function similarity of two genes. In another words, genes with similar sequence more likely interact for the purpose of carrying out similar functions.

### The relationship among genomic distance, interaction numbers, and function similarity for interacting gene pairs

**Fig. 8:**
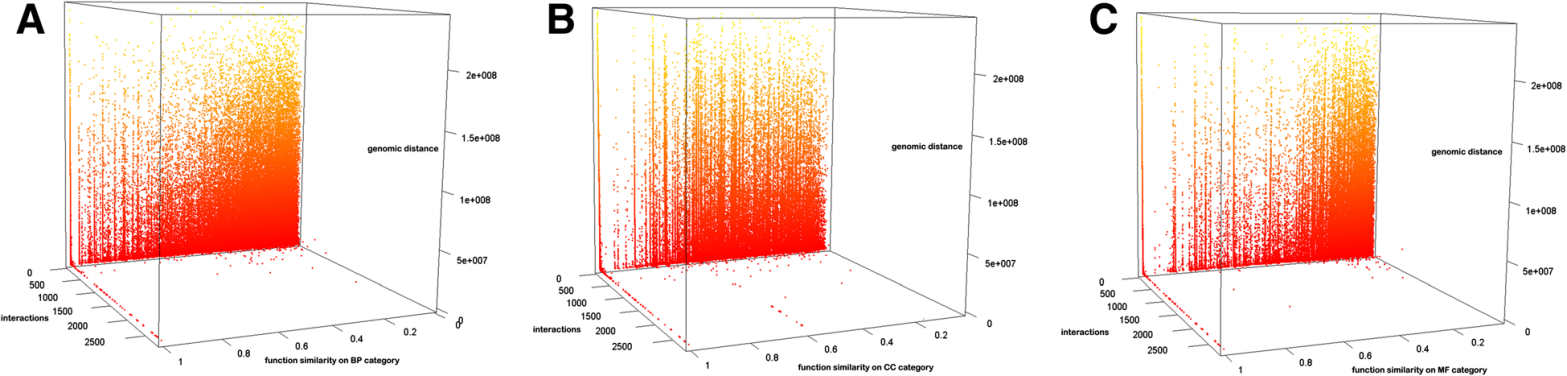
The 3D plot of genomic distance, number of interactions and the function similarity in three function categories. Figure 8**a**, **b**, and **c** represent the histogram for Biological Process, Cellular Component, and Molecular Function respectively. The yellow dots represent long genomic distances and the red ones the opposite

Figure 8 is the 3D plot of genomic distance, number of interactions and function similarity for interacting gene pairs. Since it is impossible to calculate the genomic distance between inter-chromosomal gene pairs, the analysis in Fig. 8 only considers intra-chromosomal gene-gene interactions in order to calculate the genomic distance between the genes. According to Fig. 8a and c, although the number of interactions between genes generally increases as their genomic distance decreases, most of gene pairs with short genomic distance, but small number of interactions tend to have low function similarity in terms of biological process and molecular function. According to Fig. 8b, for quite a few gene pairs with high function similarity (>0.9) in terms of cellular component, their genomic distance varies a lot when the number of interactions are small, however, when the number of interactions is large, their genomic distance is short. In order to consider the genomic distance for intra-chromosomal gene-gene interactions, we generated two new analyses by separating the gene pairs into two groups: short-range interaction pairs and long-range interaction pairs by using the median genomic distance between interacted gene pairs as threshold. The 3D plot of genomic distance, number of interactions, and function similarity for these two groups are shown in Additional file 1: Figure S15. Generally, the pattern regarding the relationships among function similarity, genomic distance and number of gene-gene interactions in Additional file 1: Figure S15 is similar to that in Fig. 8. However, one interesting finding is that the relationship between genomic distance and function similarity somewhat differ for these two groups. For the gene pairs with genomic distance longer than the median, the function similarity clearly decreases as the increasing of genomic distance (see Additional file 1: Figures S15A, S15B, and S15C), whereas no very clear such pattern has been found in gene pairs with short genomic distance (see Additional file 1: Figures S15D, S15E, S15F). For the gene pairs with shorter genomic distance, the number of gene-gene interactions has more impact on function similarity than genomic distance. Taken together, the results suggest the complementarity of the two factors in informing gene function similarity.

### Evaluation of gene function predictions based on spatial gene-gene interactions

We developed a gene function prediction method based on spatial gene-gene interaction networks, which predicts the function of a gene using the known functions of its spatially interacted neighbours (see Methods section for details). We calculated the probabilistic relationship between GO terms of a gene and the GO terms of its neighbouring genes on the spatial interaction networks constructed from the Hi-C data of the ALL B-cell. The knowledge was applied to make gene function prediction on the Call4 cell-line. We generated networks with different interaction thresholds (> = 1, 2, 3, 4, 6, 8, 10, 12, 14, 16) for the Call4 cell line. For the case of 0 threshold, which means there is no interaction between genes, our current function prediction method based on spatial gene-gene interaction cannot make any prediction. This means that our current function prediction method is limited on predicting the functions of the genes on the gene-gene interaction network, which could be expanded in the future to make function prediction using other information, such as gene sequence identity.

**Fig. 9:**
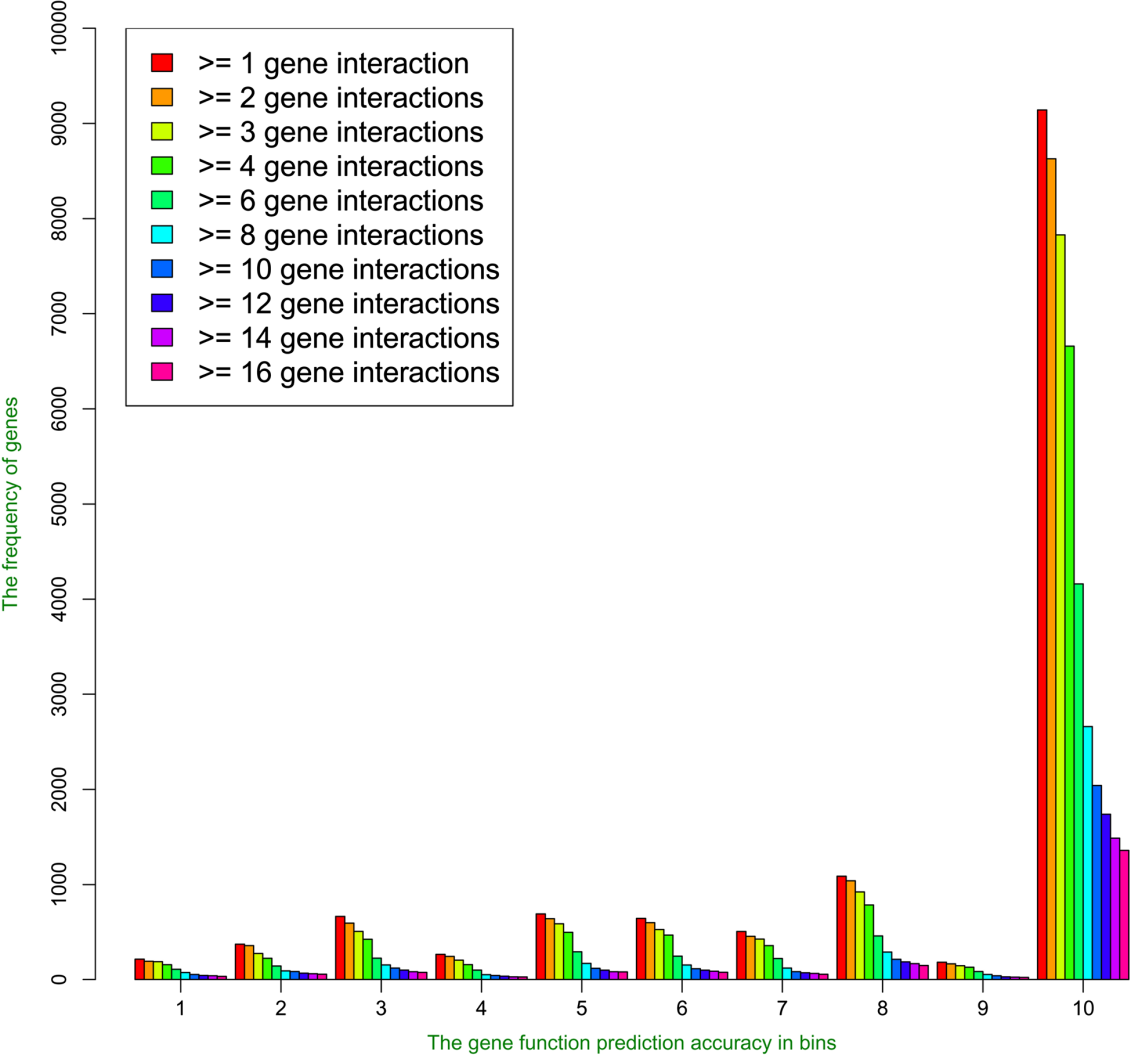
The histograms of function prediction accuracy (the maximum similarity scores between predicted GO terms and real GO terms) on the spatial gene-gene interaction networks of the Call4 cell line at different interaction thresholds. The y-axis denotes the gene frequencies and the x-axis the gene function prediction accuracies in 10 bins. 10 GO terms are selected as function prediction for each gene. The number of genes with gene function prediction accuracy in bin 10 for each gene interaction threshold separately is as follows: 9142, 8628, 7829, 6660, 4158, 2659, 2041, 1738, 1486, and 1357. The number of genes with gene function prediction accuracy in bin 1 for each gene interaction threshold separately is as follows: 214, 192, 188, 156, 108, 74, 53, 43, 40, and 33

Figure 9 illustrates the histogram of the similarities between predicted functions and true functions of the tested genes. For all the thresholds, the similarity score of the predictions for the majority of tested genes were very high (>0.9). When the interaction threshold is set to the lowest number, i.e.1, at least one highly accurate function was predicted for ~9000 genes, while much fewer genes had predictions with relative lower accuracy. This indicates that the prediction method is rather robust against the potential noise in the interaction data. As the interaction thresholds increased, the function predictions could be made for fewer genes as there were fewer interacting genes in the spatial gene interaction network. However, the percentage of genes having high accurate predictions (similarity score >0.9) is generally higher. For example, with interaction threshold 1, the number of genes having high accurate predictions (similarity score > 0.9) is 9142, and the number of genes having low accurate predictions (similarity score < 0.1) is 214; with interaction threshold 16, the number of genes having high accurate predictions (similarity score > 0.9) is 1357, and the number of genes having low accurate predictions (similarity score < 0.1) is 33.

**Fig. 10:**
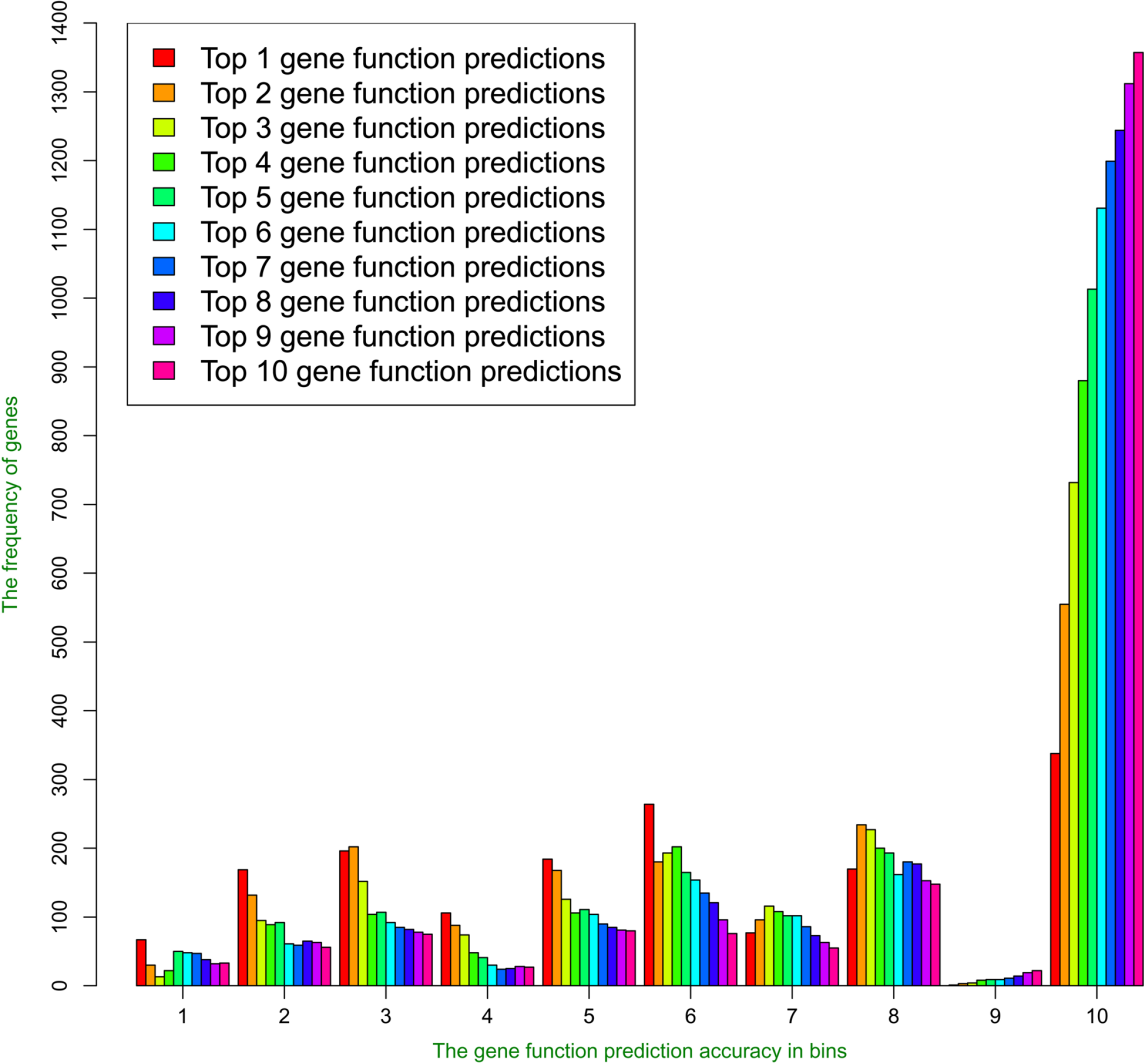
The histograms of function prediction accuracies for different numbers (1–10) of GO terms selected as predictions. The y-axis denotes the gene frequencies and the x-axis the gene function prediction accuracies in 10 bins. The number of genes with gene function prediction accuracy in bin 10 while selecting different numbers (1–10) of GO terms is as follows: 338, 555, 732, 880, 1013, 1131, 1199, 1244, 1312, and 1357. The number of genes with gene function prediction accuracy in bin 1 while selecting different numbers (1–10) of GO terms is as follows: 67, 30, 13, 22, 50, 48, 47, 38, 32, and 33

The number of GO function terms predicted for each gene also affects the sensitivity and specification of gene function prediction. Figure 10 shows the histograms of the maximum function similarity between predicted GO terms and true GO terms. Not surprisingly, as the number of GO term prediction increased, more and more genes got at least one highly similar GO function prediction.

## Conclusions

In this work, we investigated the relationship between spatial gene-gene interactions and gene function similarities. Our analyses demonstrate that genes with strong spatial interaction tend to have (nearly) the same gene function, while the weaker spatial interactions have much less correlation with gene function similarity. We also discovered that interacting genes with very high function similarity have shorter genomic distance and higher sequence identity than the rest of the interacting genes. Combining sequence identity or genomic distance with gene-gene interactions can help identify the group of interacting genes with high function similarity. The power of discriminating gene function similarity by combining spatial gene-gene interactions with sequence identity or genomic distance appears to be stronger than using each of them alone. Moreover, since the general correlation between spatial gene-gene interactions and sequence identity (or genomic distance) is rather weak in general, their stronger correlations in interacting genes with high function similarity seem to suggest that functioning together might be a reason bringing genes with highly similar functions together.

To further validate the relationship between spatial gene-gene interactions, we used the known gene function of the interacting genes of a target gene to predict its function and evaluate the prediction accuracy. Our experiment demonstrates that spatial gene-gene interactions are effective in predicting gene functions.

It is worth noting that the Hi-C data sets used in this work were generated from a population of cells rather than a single cell such that the gene-gene interaction data is an average of the spatial interactions of a population of cells whose genome conformation may vary. Furthermore, there is some noise in the data due to the experimental limitations such as variation of GC content in genomes and the biases of restriction enzymes. Taken these two factor together, it is important to normalize the interaction data to remove the noise or biases as much as possible. In the past, normalization for the Hi-C data was often done on chromosomal contact maps, where a chromosome was divided into bins of equal-length and the number of contacts between bins were calculated and normalized. However, the situation in our analysis on gene-gene interacting network is different from the normalization of chromosomal contact maps because there is no contact matrix and the lengths of genes are also different. Therefore, traditional normalization methods cannot be directly applied to our gene-gene interaction data. So, we applied a simple, new normalization approach by selecting different interaction thresholds of contacts in order to get similar topology of networks for the four cells/cell lines. Although this cross-dataset normalization approach is not ideal, it can still retain most of the pattern in the data, leading to valuable findings regarding gene function similarity. In the future, better methods of removing biases in gene-gene interaction data need to be developed and applied to improve the analysis of gene function similarity.

Moreover, more and more Hi-C data with better quality than the four datasets used in this work have been available. We will apply the approach developed in this work to the new datasets to further study the function similarity between interacted genes in the near future.

## Methods

### Calculation of gene function similarity between two genes

We used the Gene Ontology (GO) terms [17] to describe the function of a gene in three categories: Molecular Function (MF), Biological Process (BP) and Cellular Component (CC). We applied the online tool G-SESAME [20] and the python package FastSemSim [21] to calculate the functional similarity score between any two GO terms. The annotated functions of the human genes were retrieved from the Uniprot database [22]. We used the maximum function similarity score between the GO terms of two genes as the measure of the function similarity between them when we assessed the function similarity of interacted and non-interacting gene pairs.

### Construction of genome-wide spatial gene-gene interaction networks

We downloaded the gene information (the start and end positions of the genes) of the human genome (build 36.3) from the NCBI website. We only considered the “GENE” entries without using other entries, such as “PSEUDO”, “RNA”, “CDS” and “UTR”. Based on the gene definitions, we constructed spatial gene-gene interaction networks from the Hi-C data of the Primary human B-acute lymphoblastic leukemia (ALL), the MHH-CALL-4 B-ALL cell line (CALL4), and the follicular lymphoma cell-line (RL) sequenced using an Illumina HiSeq 2000 [10], as well as that of the normal human B-cell line (GM06990) [9].

### Calculation of sequence identity

The dynamic programming technique is used to calculate the sequence identity of two protein sequences of a gene pair. Given two protein sequences: *X* = (*x*_1_, *x*_2_, … , *x*_*m*_) and *Y* = (*y*_1_, *y*_2_, … , *y*_*n*_), we define the i_th_ prefix of X as *X*_*i*_ = (*x*_1_, *x*_2_, … , *x*_*i*_), i is in the range between 1 and m. The longest continuous / non-continuous common subsequence (LCS) of these two sequences (*LCS*(*X*, *Y*)) is the longest subsequence which exists in both sequences. We define *c*[*i*, *j*] to be the length of *LCS*(*X*_*i*_, *Y*_*j*_). The following recursive formula is used for calculating the length of *LCS*(*X*_*i*_, *Y*_*j*_): [23].

$$ c\left[i,j\right] = \left\{\kern0.5em \begin{array}{c}0,\kern10.7em  if\ i=0\kern0.5em  or\ j=0\hfill \\ {}c\left[i-1,j-1\right] + 1\kern5.61em  if\left(i,j>0\kern0.5em  and\ {x}_i={y}_j\right)\hfill \\ {} \max \left(c\left[i,j-1\right],c\left[i-1,j\right]\right)\kern2.5em  if\left(i,j>0\kern0.5em  and\kern0.5em {x}_i\ne {y}_j\right)\hfill \end{array}\right. $$

A m*n matrix is used to for storing *c*[*i*, *j*]. *c*[*m*, *n*] contains the length of *LCS*(*X*, *Y*). We calculate the sequence identity of two protein sequences as *LCS*(*X*, *Y*) divided by the maximum sequence length of X and Y.

To make comparison, we also apply Needleman-Wunsch algorithm to align two sequences using BLOSUM62 as a substitution matrix, and calculate the sequence identity as the percentage of aligned part between these two sequences.

### Gene function prediction based on spatial gene-gene interaction networks

The gene function prediction method has Five steps: (1) calculating the probability of a GO term (GO_1_) for a gene given a known GO term (GO_2_) of its neighboring gene, i.e., P(a gene has GO_1_ | the gene’s neighbor has GO_2_), based on the entire interaction networks of the ALL B-cell; (2) For each gene on the interaction network of the Call4 cell line, randomly selecting one of its neighboring gene having function annotations; (3) Obtaining the GO terms of the selected neighboring gene; (4) For each GO term (G_i_) of the neighboring gene, calculating the probability of other GO terms (G_j_) for the target gene according to the conditional probability P(G_j_ | G_i_) pre-computed in Step (1); and (5) summing up the probabilities of each GO term inferred for the target gene into frequencies and ranking the GO terms based on their frequencies as the predictions for the target gene.

Once one or more GO terms are predicted for a gene, we use FastSemSim to compute the similarity between each predicted GO term and each of the real GO term of the gene. The maximum similarity between a predicted GO term and a real GO term is considered as the accuracy (i.e. similarity score) of the prediction.

## Additional file

Additional file 1:
**Supplementary figures and tables.** (DOCX 4953 kb)
