# Supplementary material for: Deciphering the association between gene function and spatial gene-gene interactions in 3D human genome conformation
Source: BMC Genomics. 2015 Oct 28;16:880. doi: 10.1186/s12864-015-2093-0 (PMC4625479; doi:10.1186/s12864-015-2093-0)
Supplement: Additional file 1: — Supplementary figures and tables. (DOCX 4953 kb) [file 12864_2015_2093_MOESM1_ESM.docx]

**Supplementary Materials**

**Figure S1** – The number of nodes in the largest cluster with different interaction threshold of all four cell lines. The x-axis is the interaction threshold to parse the gene-gene interaction network, and the y-axis shows the number of nodes for the largest cluster in the gene-gene interaction network.


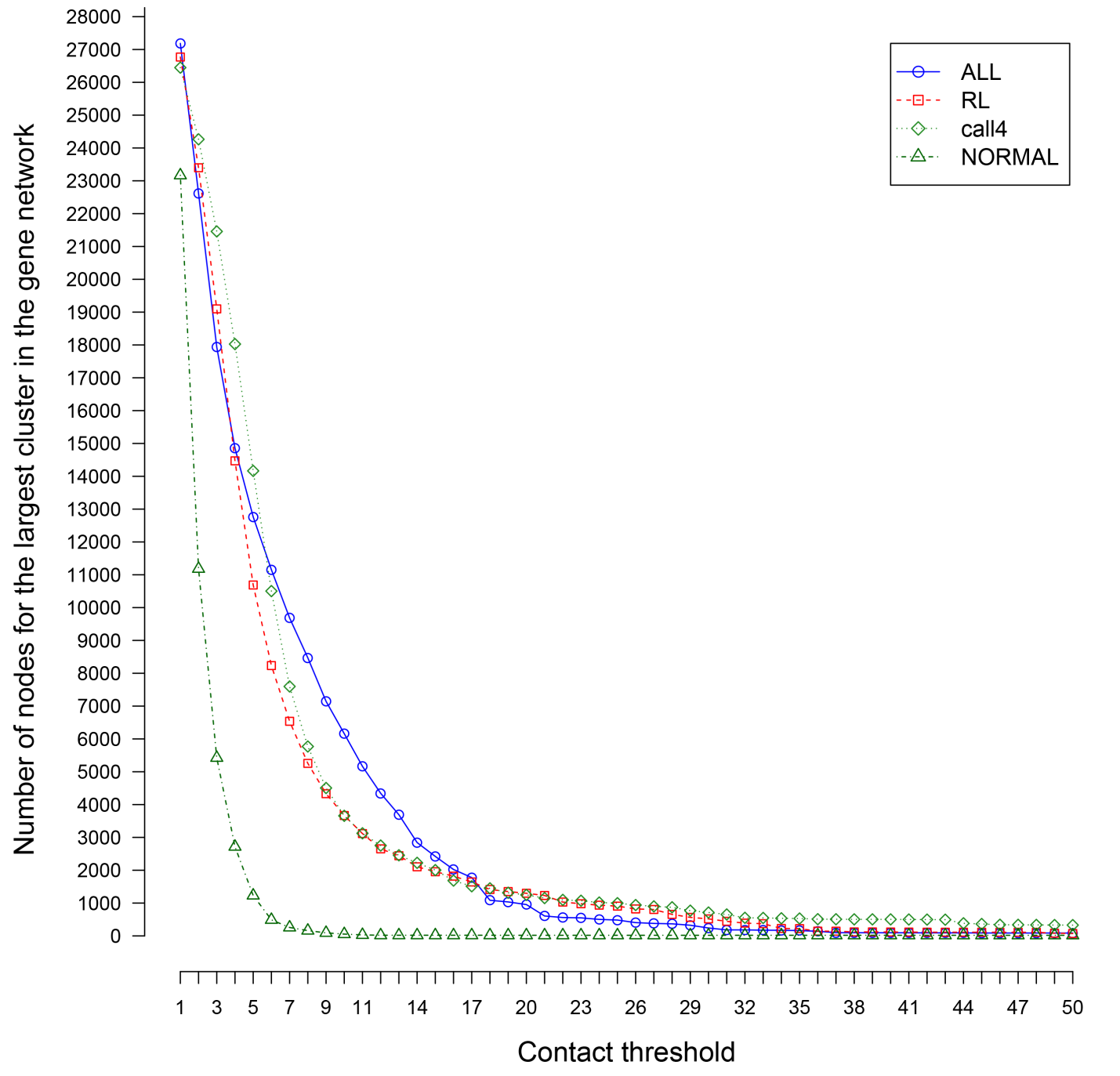


**Figure S2** – The average number of interactions between substantially interacting gene pairs of Call4 cell line, normal B-cell, and RL cell line. Figure S2 (A), (B), and (C) is generated for Call4 cell line of each function category, Figure S2 (D), (E), and (F) is generated for normal B cell of each function category, Figure S2 (H), (I), and (J) is generated for RL cell line for each function category.

(A) (B) (C)


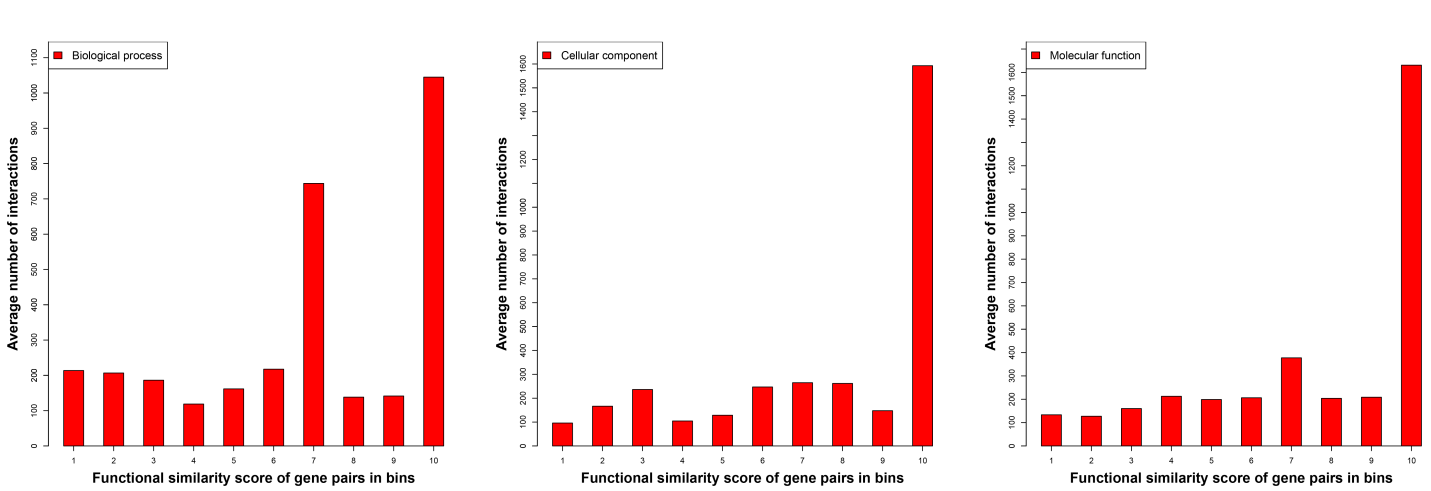


(D) (E) (F)


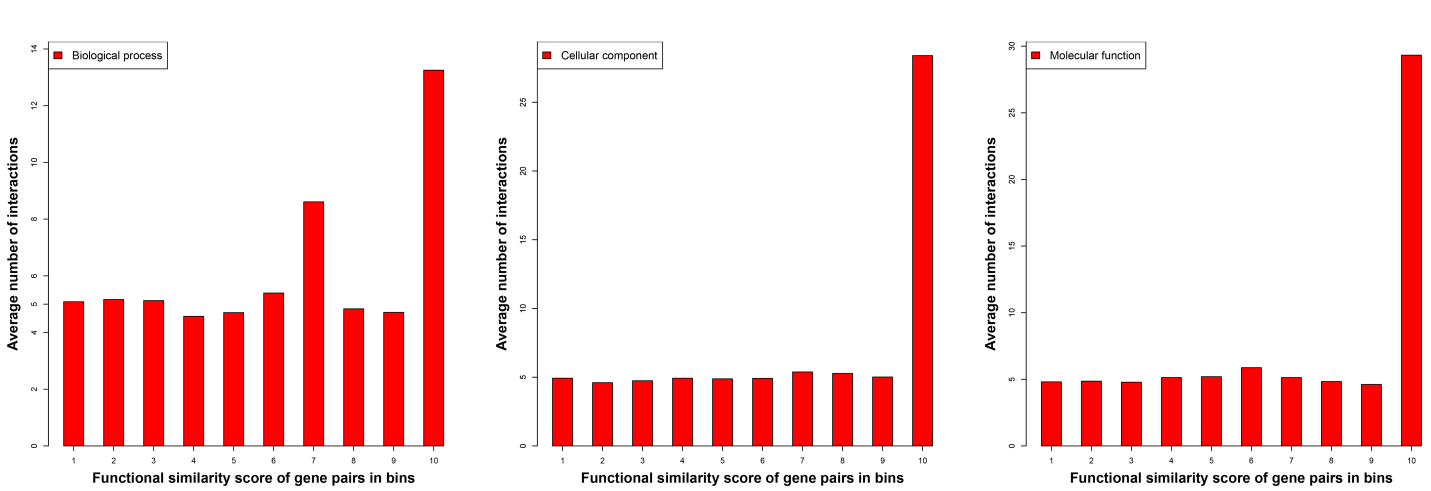


(H) (I) (J)


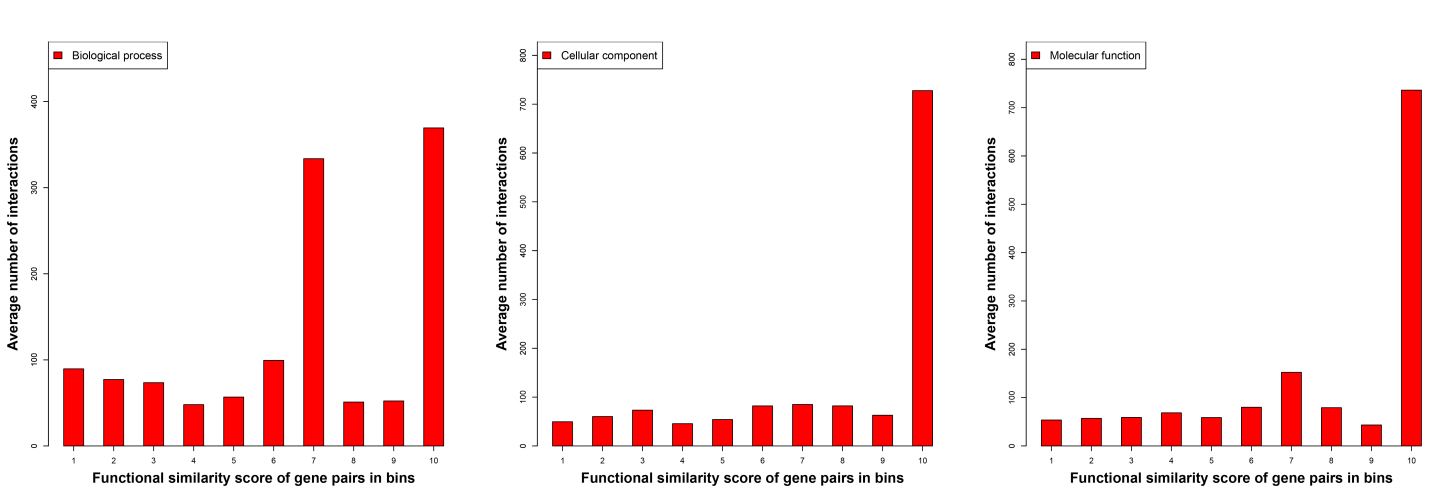


**Figure S3** – The boxplot of the numbers of interactions in each functional similarity bin calculated for all three function categories for primary tumor B-cells (ALL) cell at 18 interaction thresholds. The R statistical package was used to generate the plots. The dots in the figure are outliers identified by the R program. The five-point statistical summary (quantiles at 0%, 25%, 50%, 75%, and 100%) of all bins is listed on the right. Figure S3(A), Figure S3(B), and Figure S3(C) is the boxplot for biological process (BP), cellular component (CC), and molecular function (MF) function categories separately.

(A)

| 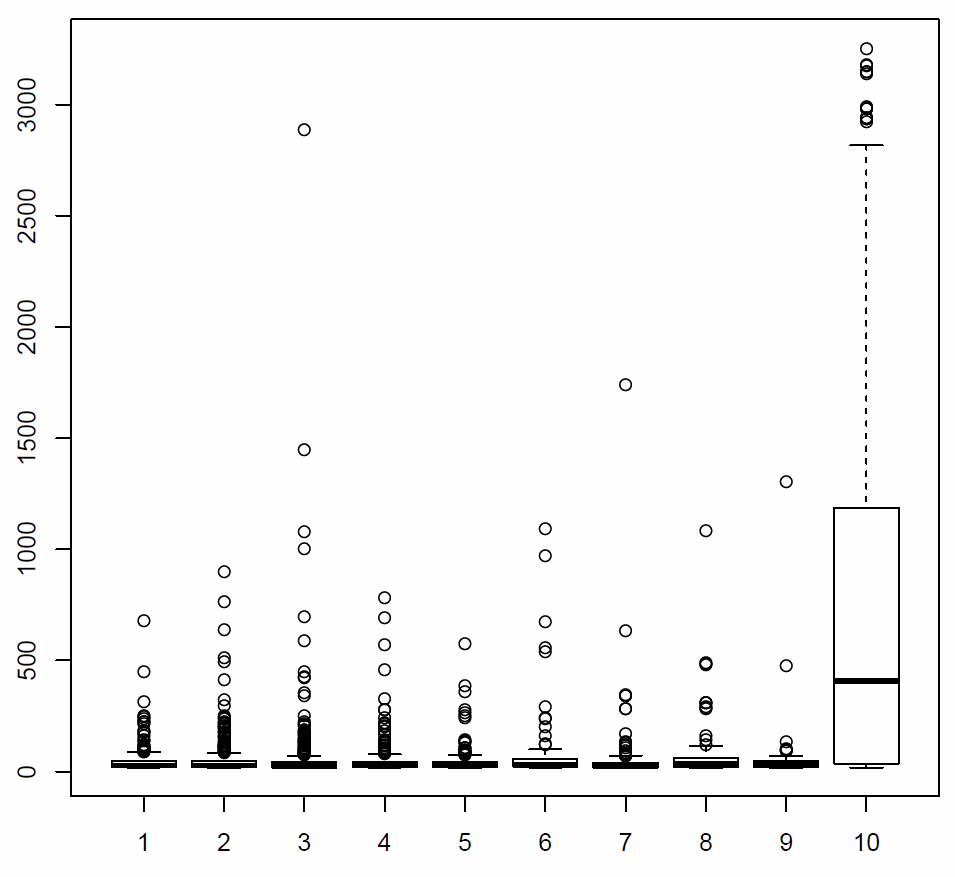 | Bins | 0% | 25% | 50% | 75% | 100% |
| --- | --- | --- | --- | --- | --- | --- |
|  | 1 | 18 | 21 | 28 | 47.75 | 681 |
|  | 2 | 18 | 22 | 28 | 47 | 902 |
|  | 3 | 18 | 21 | 26 | 42.75 | 2888 |
|  | 4 | 18 | 21 | 27 | 45 | 782 |
|  | 5 | 18 | 21 | 28 | 43 | 574 |
|  | 6 | 18 | 23 | 29 | 56 | 1095 |
|  | 7 | 18 | 20.75 | 26 | 41 | 1741 |
|  | 8 | 18 | 23 | 34 | 59.75 | 1083 |
|  | 9 | 18 | 22 | 31.5 | 47.75 | 1303 |
|  | 10 | 18 | 36 | 407 | 1187 | 3254 |

(B)

| 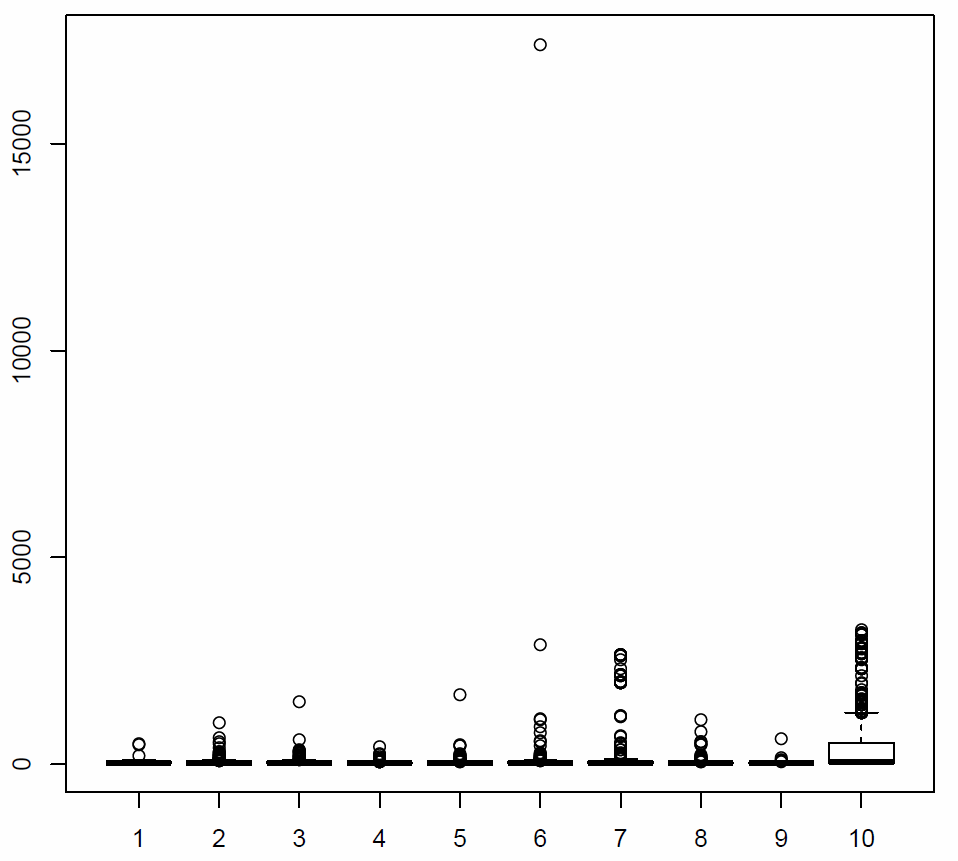 | Bins | 0% | 25% | 50% | 75% | 100% |
| --- | --- | --- | --- | --- | --- | --- |
|  | 1 | 18 | 21 | 29 | 60 | 494 |
|  | 2 | 18 | 21 | 28 | 51 | 1020 |
|  | 3 | 18 | 21 | 30 | 60 | 1517 |
|  | 4 | 18 | 21 | 26 | 39 | 427 |
|  | 5 | 18 | 21 | 27 | 43 | 1679 |
|  | 6 | 18 | 21 | 28 | 52 | 17401 |
|  | 7 | 18 | 23 | 31 | 68.75 | 2663 |
|  | 8 | 18 | 22 | 28 | 43.75 | 1083 |
|  | 9 | 18 | 23 | 31 | 39.75 | 635 |
|  | 10 | 18 | 25 | 51 | 516 | 3254 |

(C)

| 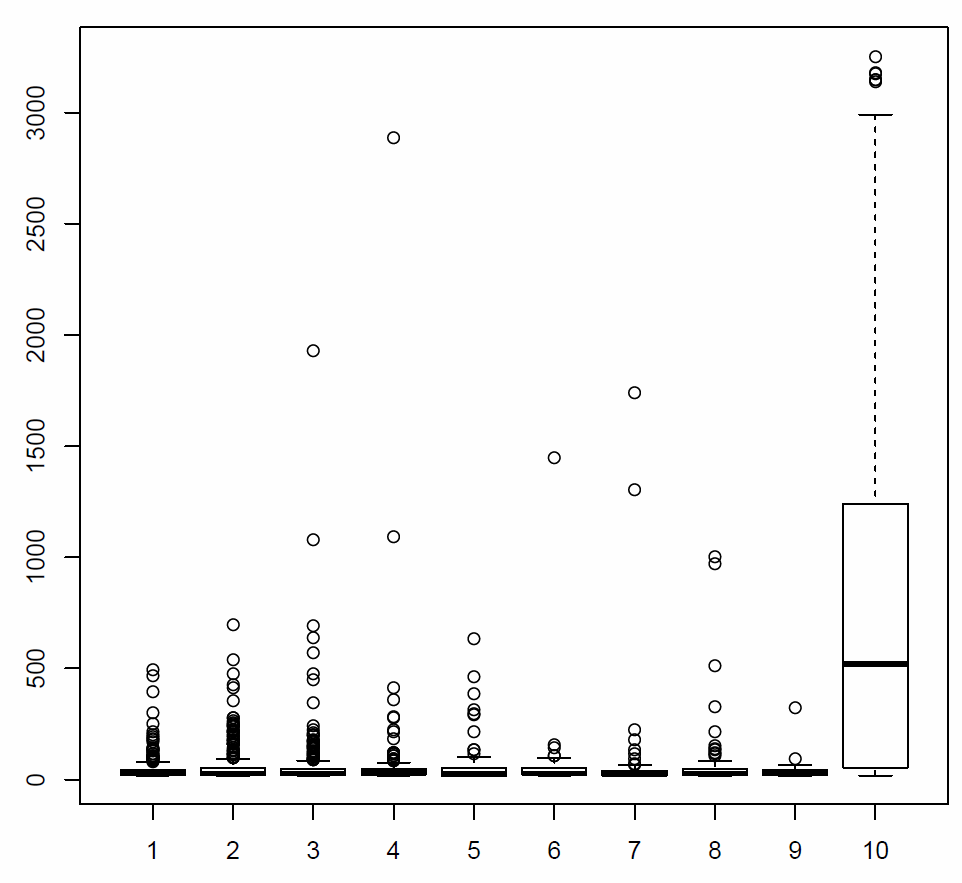 | Bins | 0% | 25% | 50% | 75% | 100% |
| --- | --- | --- | --- | --- | --- | --- |
|  | 1 | 18 | 22 | 28 | 45 | 494 |
|  | 2 | 18 | 21 | 28 | 52 | 697 |
|  | 3 | 18 | 21 | 27 | 47 | 1928 |
|  | 4 | 18 | 23 | 29 | 46.75 | 2888 |
|  | 5 | 18 | 21 | 26 | 53 | 635 |
|  | 6 | 18 | 23.25 | 28 | 53 | 1447 |
|  | 7 | 18 | 21 | 26 | 39 | 1741 |
|  | 8 | 18 | 21 | 27 | 47 | 1004 |
|  | 9 | 18 | 20 | 30 | 45 | 322 |
|  | 10 | 18 | 54 | 519 | 1240 | 3254 |

**Figure S4** – The boxplot of genomic distances of gene pairs with each functional similarity score in three categories (A: Biological Process, B: Cellular Component, C: Molecular Function) on spatial gene-gene interaction networks of the ALL B-cell at interaction threshold 18. X-axis denotes the function similarity scores in 10 bins and Y-axis the genomic distance.

(A)

| 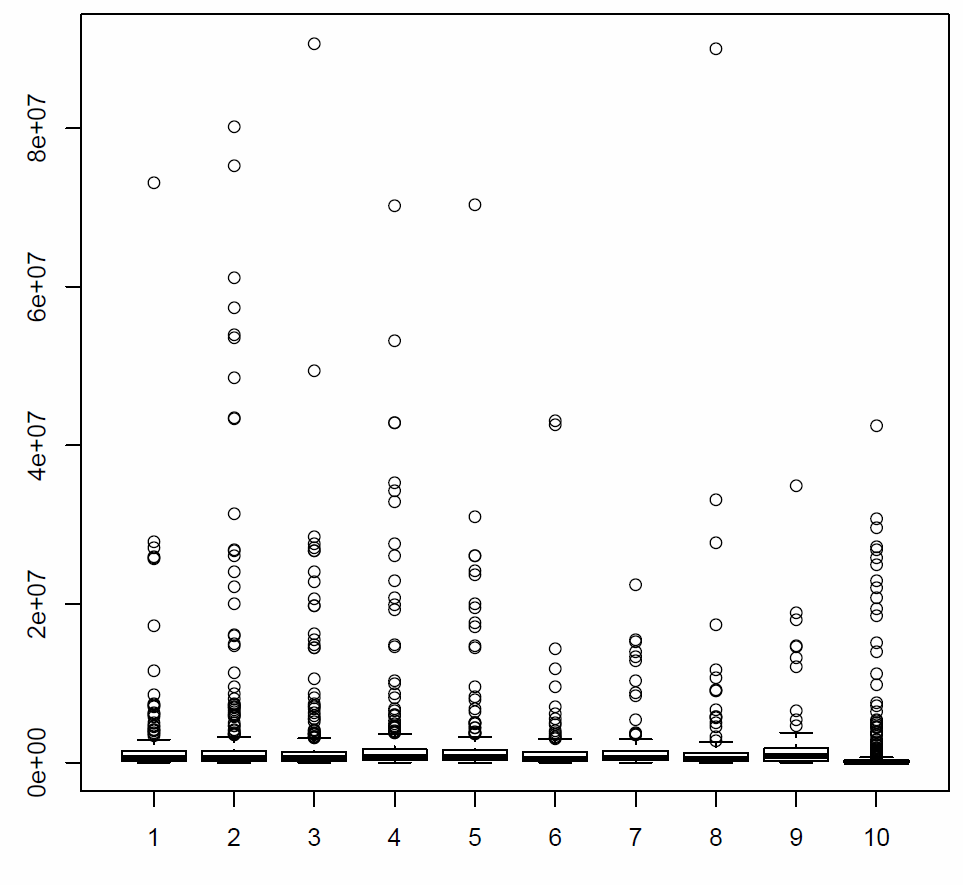 | Bins | 0% | 25% | 50% | 75% | 100% |
| --- | --- | --- | --- | --- | --- | --- |
|  | 1 | 44 | 231001.8 | 574281 | 1459668 | 73151895 |
|  | 2 | 5293 | 217131 | 586980.5 | 1459254.8 | 80166696 |
|  | 3 | 745 | 205739 | 564475.5 | 1386700 | 90704474 |
|  | 4 | 47 | 288661 | 689205 | 1666646 | 70294057 |
|  | 5 | 1 | 282446 | 776905 | 1630542 | 70412342 |
|  | 6 | 907 | 187327 | 492075 | 1322094 | 43086717 |
|  | 7 | 7924 | 307300 | 653589.5 | 1499737.5 | 22498017 |
|  | 8 | 1 | 165842 | 469931 | 1185667 | 90034156 |
|  | 9 | 14296 | 230937.5 | 812523 | 1781547 | 34928341 |
|  | 10 | 0 | 29545.75 | 72312 | 289600 | 42544913 |

(B)

| 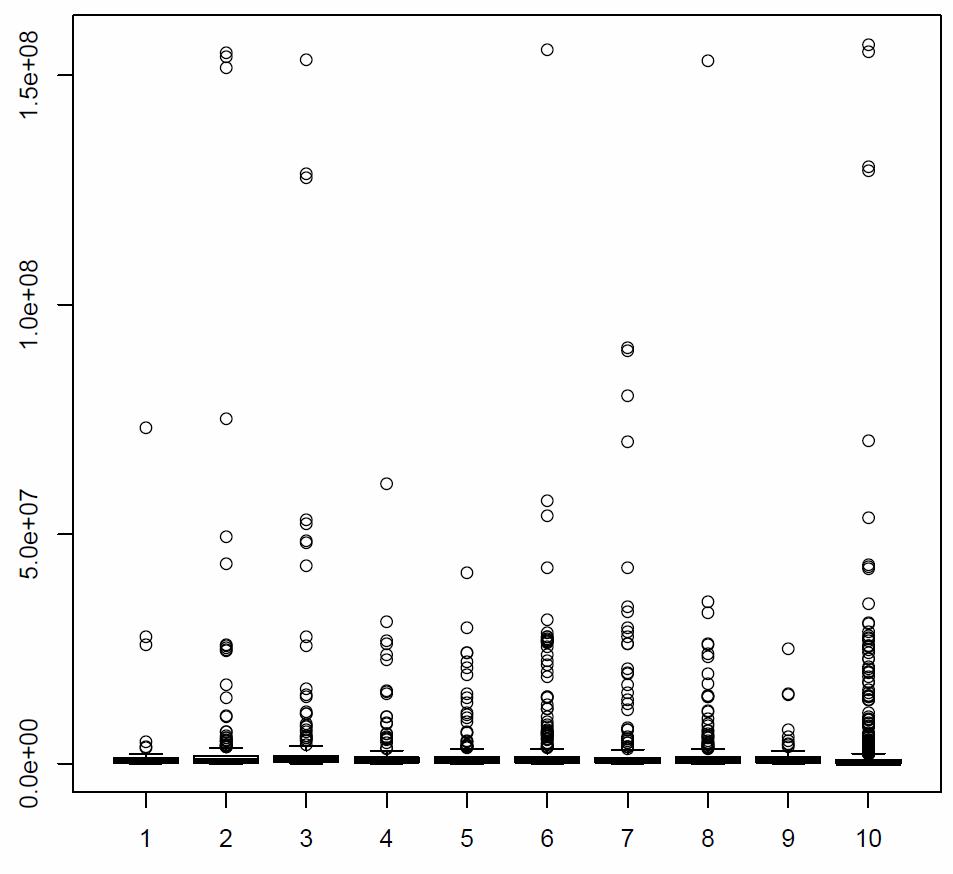 | Bins | 0% | 25% | 50% | 75% | 100% |
| --- | --- | --- | --- | --- | --- | --- |
|  | 1 | 8147 | 143033 | 458991 | 1332879 | 73151895 |
|  | 2 | 47 | 190509.5 | 532766.5 | 1585653 | 154973245 |
|  | 3 | 741 | 267511 | 741632 | 1760540 | 153429407 |
|  | 4 | 1030 | 202292.5 | 590207.5 | 1352684.2 | 61111832 |
|  | 5 | 0 | 225190 | 609214 | 1480615 | 41688046 |
|  | 6 | 5750 | 239285.5 | 575341 | 1472167.5 | 155632268 |
|  | 7 | 1 | 158569 | 537812 | 1323626 | 90704474 |
|  | 8 | 11 | 237996.5 | 657657 | 1461791.5 | 153314272 |
|  | 9 | 17574 | 264864 | 498381 | 1434718 | 25092359 |
|  | 10 | 0 | 53030.5 | 201949 | 888695.8 | 156795569 |

(C)

| 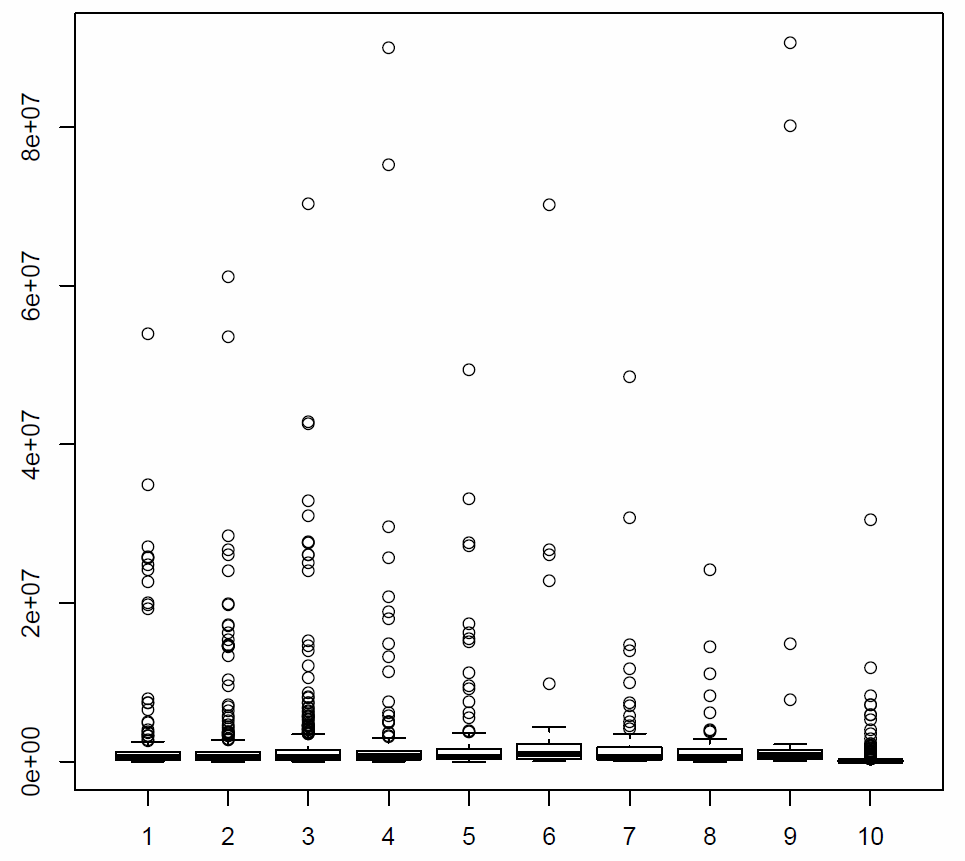 | Bins | 0% | 25% | 50% | 75% | 100% |
| --- | --- | --- | --- | --- | --- | --- |
|  | 1 | 47 | 204838.2 | 565609 | 1172031 | 53970273 |
|  | 2 | 1 | 225050.5 | 557269 | 1231900.5 | 61111832 |
|  | 3 | 3149 | 200518 | 557683 | 1513474 | 70412342 |
|  | 4 | 5293 | 262041.5 | 701091 | 1362662 | 90034156 |
|  | 5 | 11881 | 285321.5 | 627307 | 1633636.5 | 49493177 |
|  | 6 | 24236 | 294903 | 937751 | 2211068 | 70294057 |
|  | 7 | 48133 | 260694 | 639522 | 1814206 | 48579796 |
|  | 8 | 4690 | 227629.5 | 661865 | 1564025.2 | 24215903 |
|  | 9 | 34623 | 287926.8 | 838367.5 | 1373421.8 | 90704474 |
|  | 10 | 0 | 27653.25 | 64167.5 | 146016 | 30533229 |

**Figure S5** – The box plots for the sequential genomic distance in each functional similarity bin for each function category (A: Biological Process, B: Cellular Component, C: Molecular Function) of randomly selected non-interacting gene pairs. Figure S5(A), Figure S5(B), and Figure S5(C) is the boxplot for biological process (BP), cellular component (CC), and molecular function (MF) function categories separately.

(A)

| 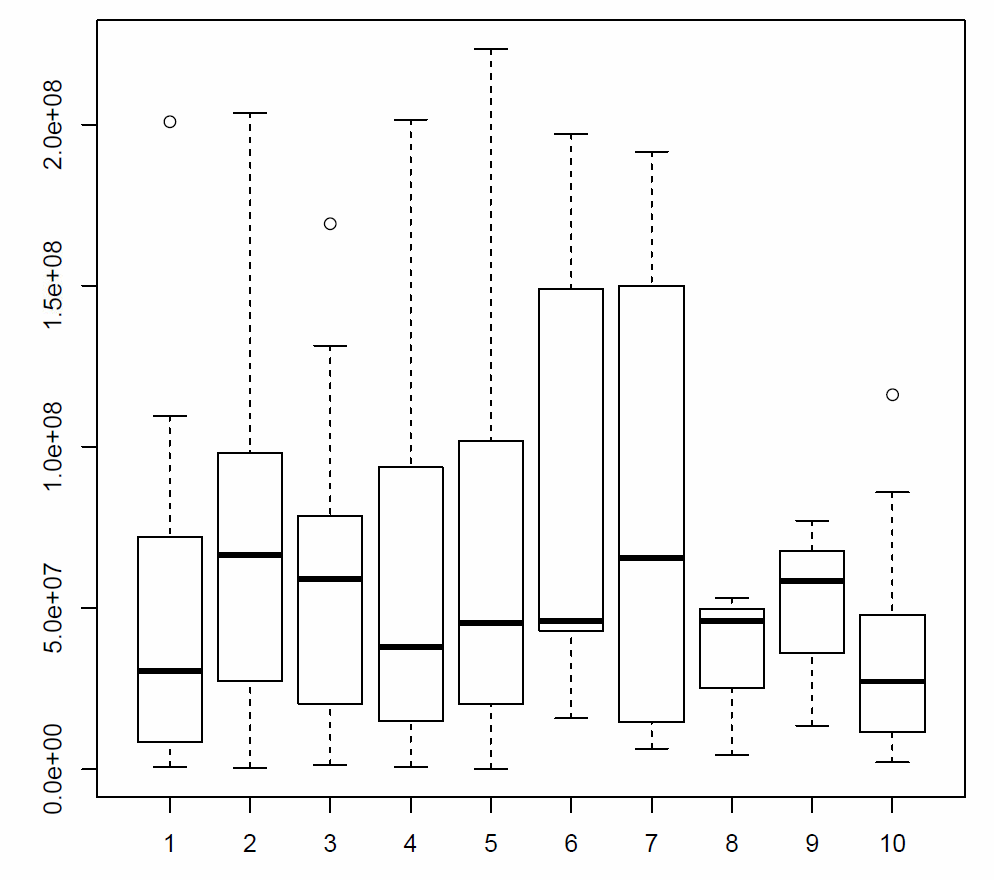 | Bin | 0% | 25% | 50% | 75% | 100% |
| --- | --- | --- | --- | --- | --- | --- |
|  | 1 | 734582 | 8293227 | 30483552 | 72002028 | 200929428 |
|  | 2 | 415328 | 28284921 | 66562110 | 94749786 | 203733880 |
|  | 3 | 1317881 | 20344099 | 59094039 | 78119328 | 169199654 |
|  | 4 | 709602 | 15435828 | 37925880 | 91413618 | 201554893 |
|  | 5 | 159409 | 23575738 | 45276014 | 97933705 | 223401331 |
|  | 6 | 15738920 | 43272636 | 46051946 | 125865678 | 197107395 |
|  | 7 | 6345593 | 18580531 | 65582524 | 129279331 | 191598709 |
|  | 8 | 4327960 | 25176042 | 46024125 | 49582658 | 53141192 |
|  | 9 | 13515120 | 35912300 | 58309481 | 67720802 | 77132122 |
|  | 10 | 2097669 | 11380676 | 27175337 | 47883632 | 116213209 |

(B)

| 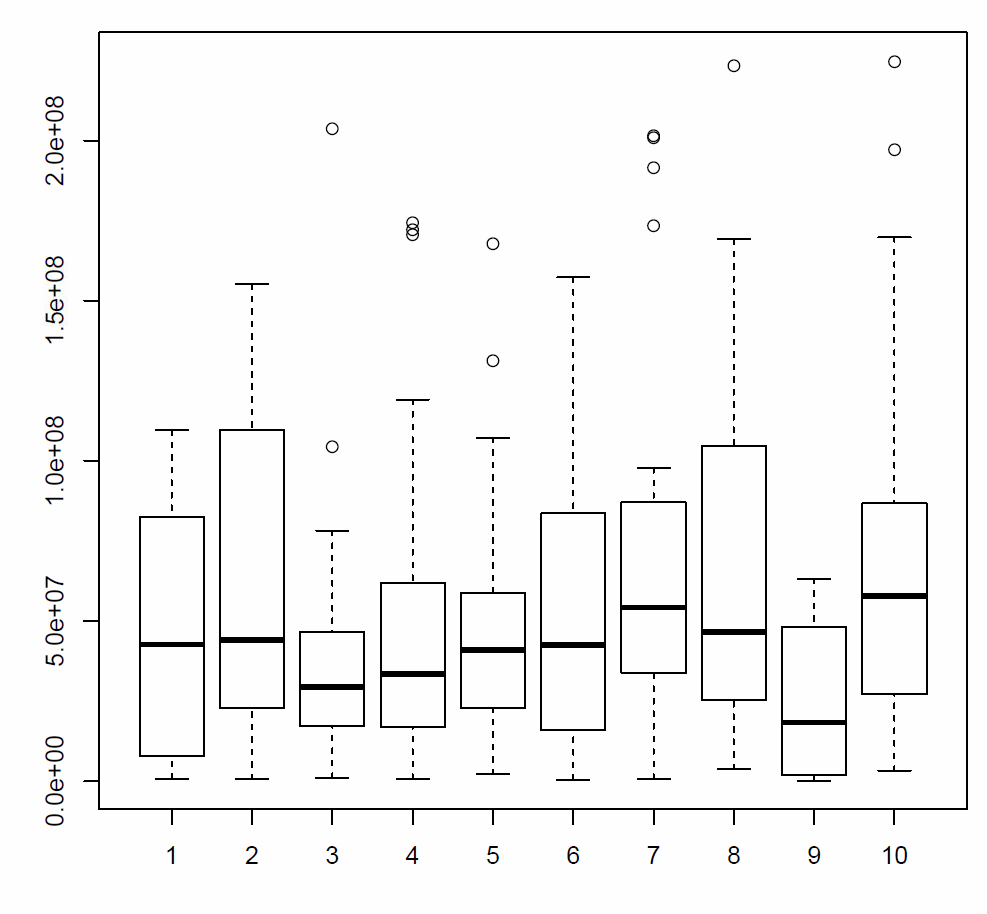 | Bin | 0% | 25% | 50% | 75% | 100% |
| --- | --- | --- | --- | --- | --- | --- |
|  | 1 | 734582 | 9585436 | 42621796 | 74683966 | 109608409 |
|  | 2 | 540623 | 26589840 | 44114706 | 101666340 | 155204556 |
|  | 3 | 984427 | 17131222 | 29384340 | 46650600 | 203733880 |
|  | 4 | 665527 | 16845466 | 33522255 | 62008950 | 174487231 |
|  | 5 | 2077818 | 22899377 | 41017220 | 58675847 | 167944984 |
|  | 6 | 415328 | 15964920 | 42411722 | 83564936 | 157272153 |
|  | 7 | 709602 | 35469981 | 54227387 | 81675672 | 201554893 |
|  | 8 | 3707940 | 25403117 | 46570749 | 104519215 | 223401331 |
|  | 9 | 159409 | 2649936 | 18335376 | 40701850 | 63235475 |
|  | 10 | 3227199 | 27175337 | 57717537 | 86784278 | 224768327 |

(C)

| 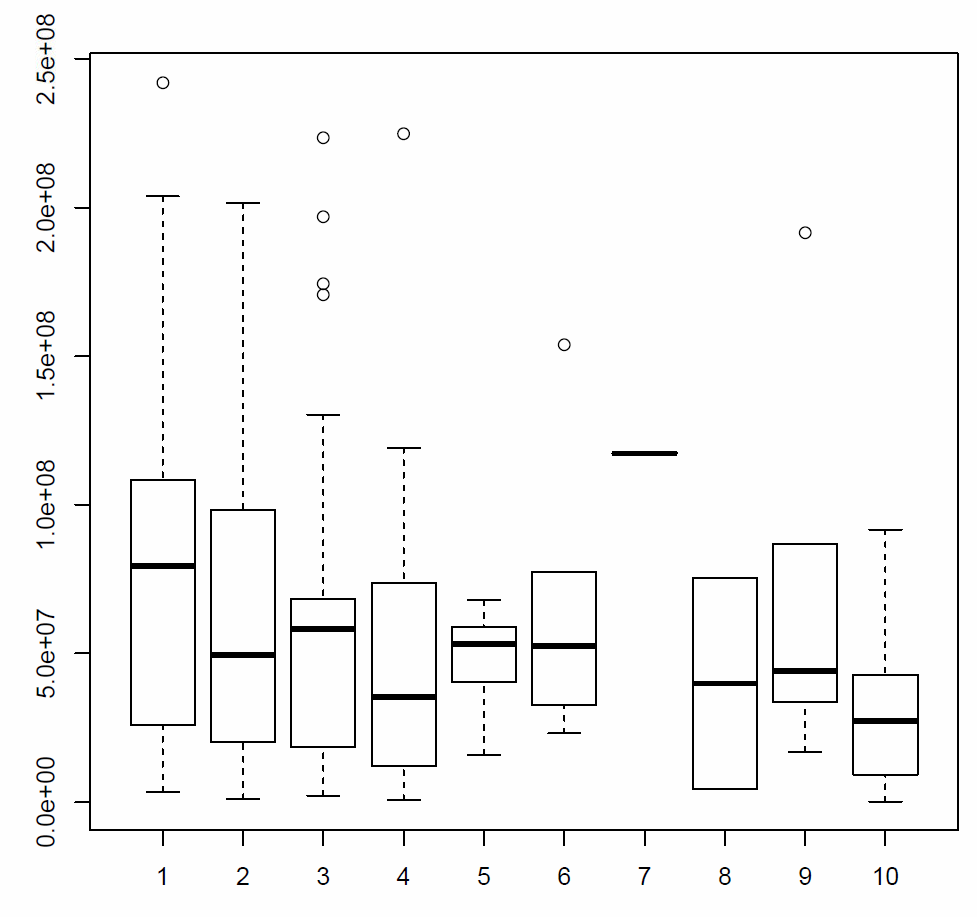 | Bin | 0% | 25% | 50% | 75% | 100% |
| --- | --- | --- | --- | --- | --- | --- |
|  | 1 | 3480111 | 26044392 | 79315634 | 108391942 | 242148867 |
|  | 2 | 984427 | 22138918 | 49535866 | 95619654 | 201554893 |
|  | 3 | 2162103 | 18474444 | 58165472 | 68241537 | 223401331 |
|  | 4 | 709602 | 12458401 | 35416146 | 58370316 | 224768327 |
|  | 5 | 15964920 | 40411241 | 53141192 | 59042213 | 67855502 |
|  | 6 | 23138712 | 32758308 | 52573076 | 77516546 | 154070091 |
|  | 7 | 117316472 | 117316472 | 117316472 | 117316472 | 117316472 |
|  | 8 | 4327960 | 22088038 | 39848116 | 57608194 | 75368272 |
|  | 9 | 16845466 | 33640628 | 44128496 | 86784278 | 191598709 |
|  | 10 | 159409 | 9277468 | 27175337 | 42742720 | 91690252 |

**Figure S6** – The average sequence identity of substantially interacting genes in each functional similarity bin in three GO function categories (Biological Process, Cellular Component, and Molecular Function) for the primary tumor B-cells (ALL). The sequence identity of Figure S6 (D), (E), (F) is calculated by Needleman-Wunsch algorithm for three GO function categories respectively.

(A) (B) (C)


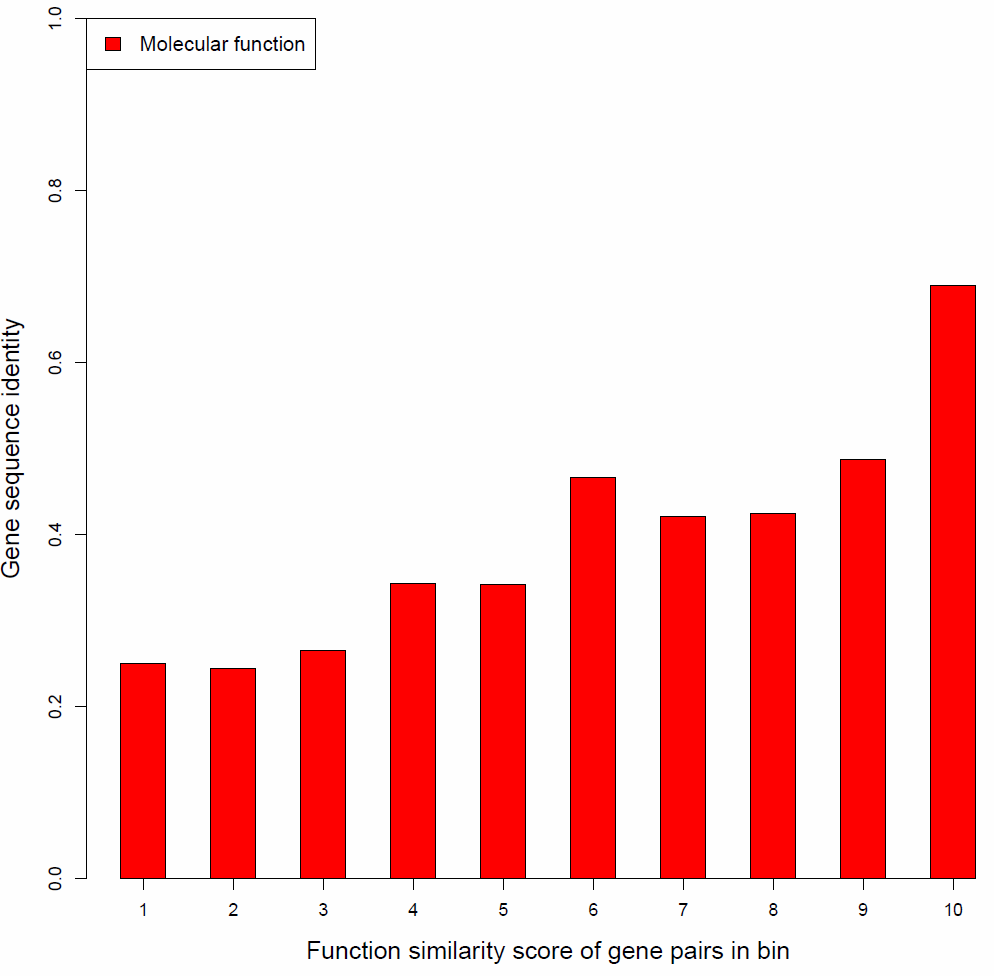

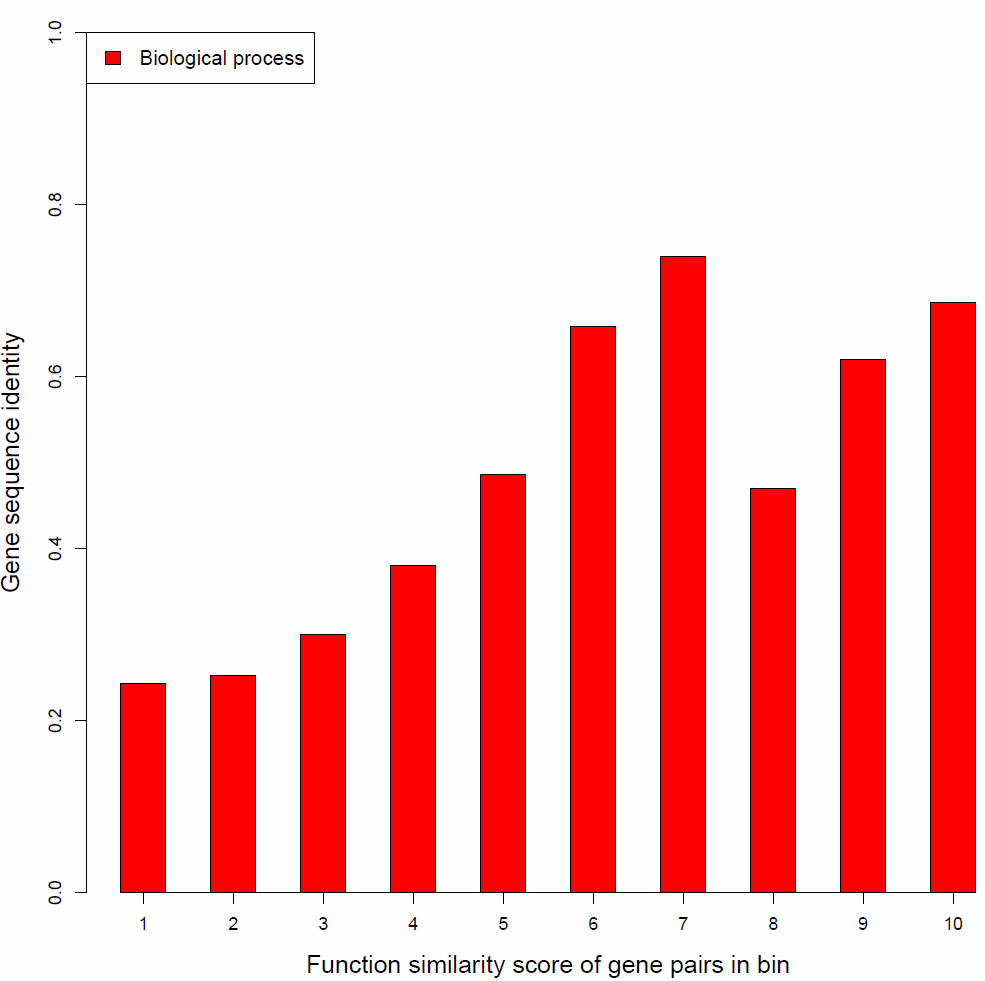

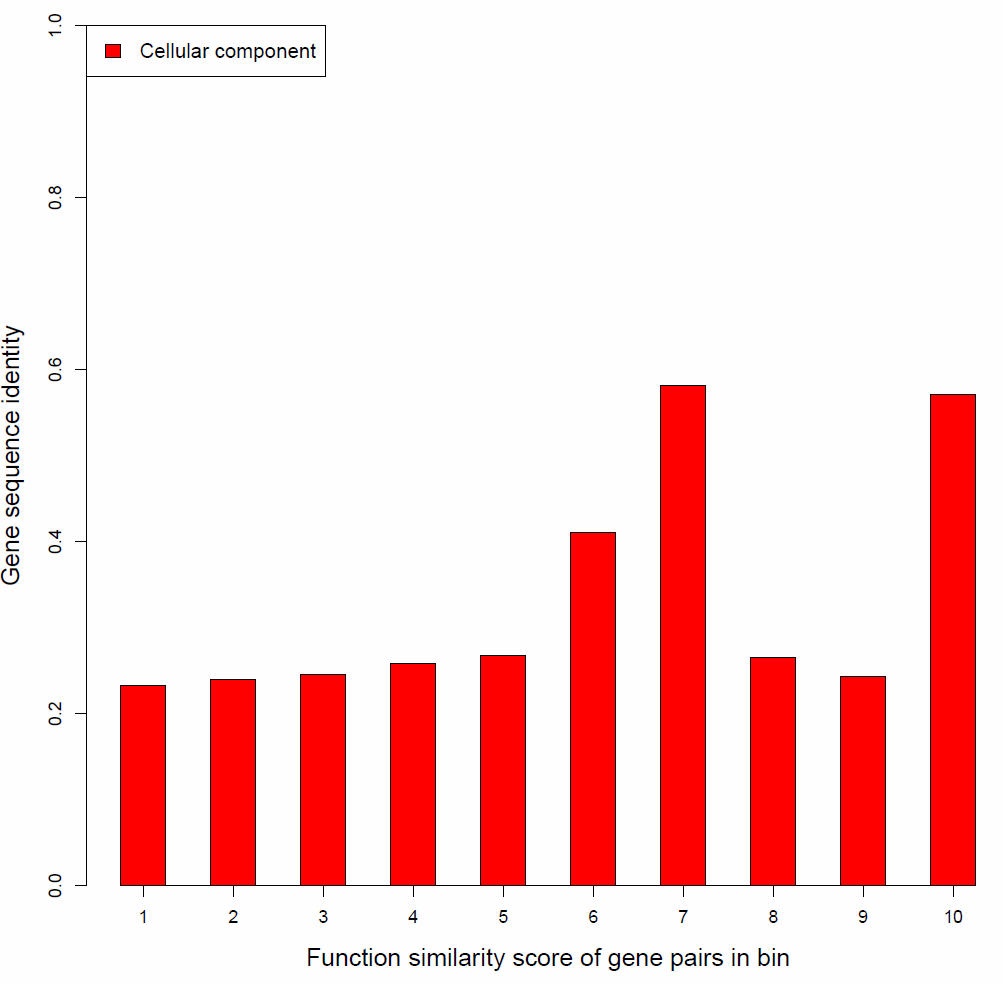


(D) (E) (F)


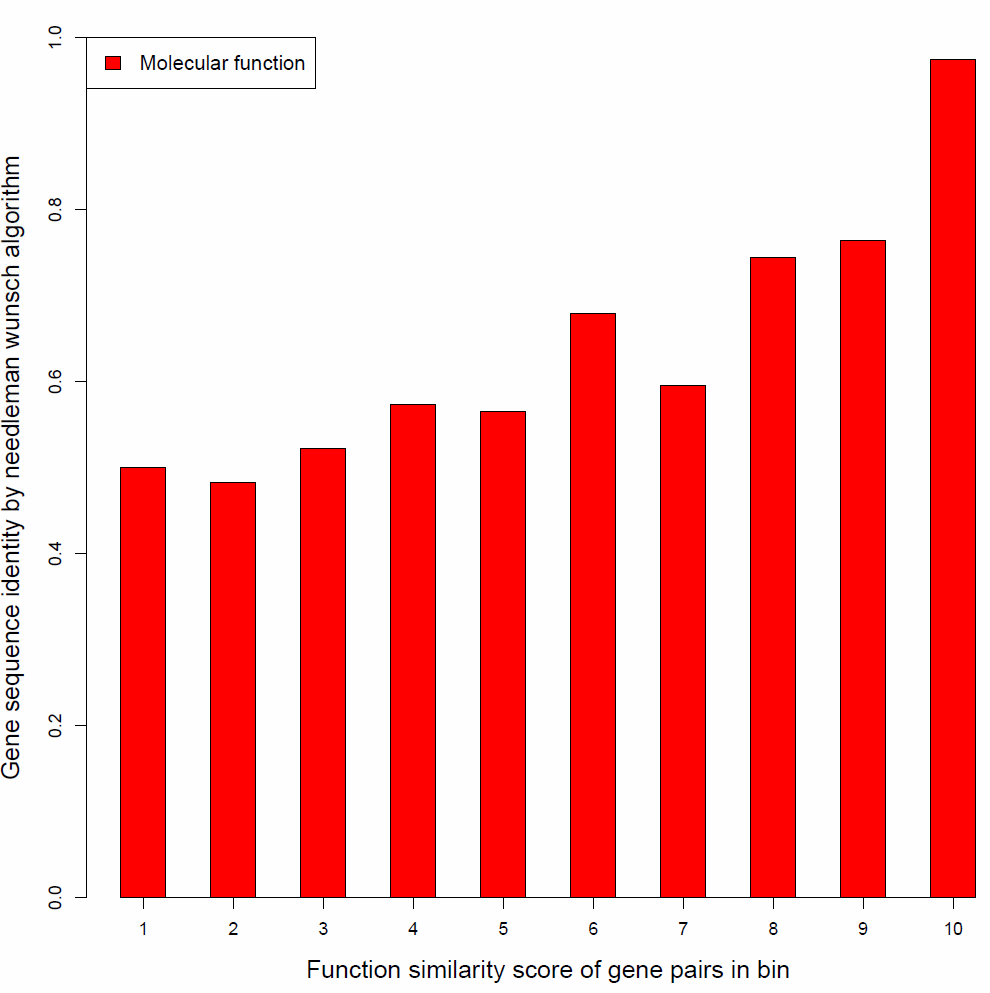

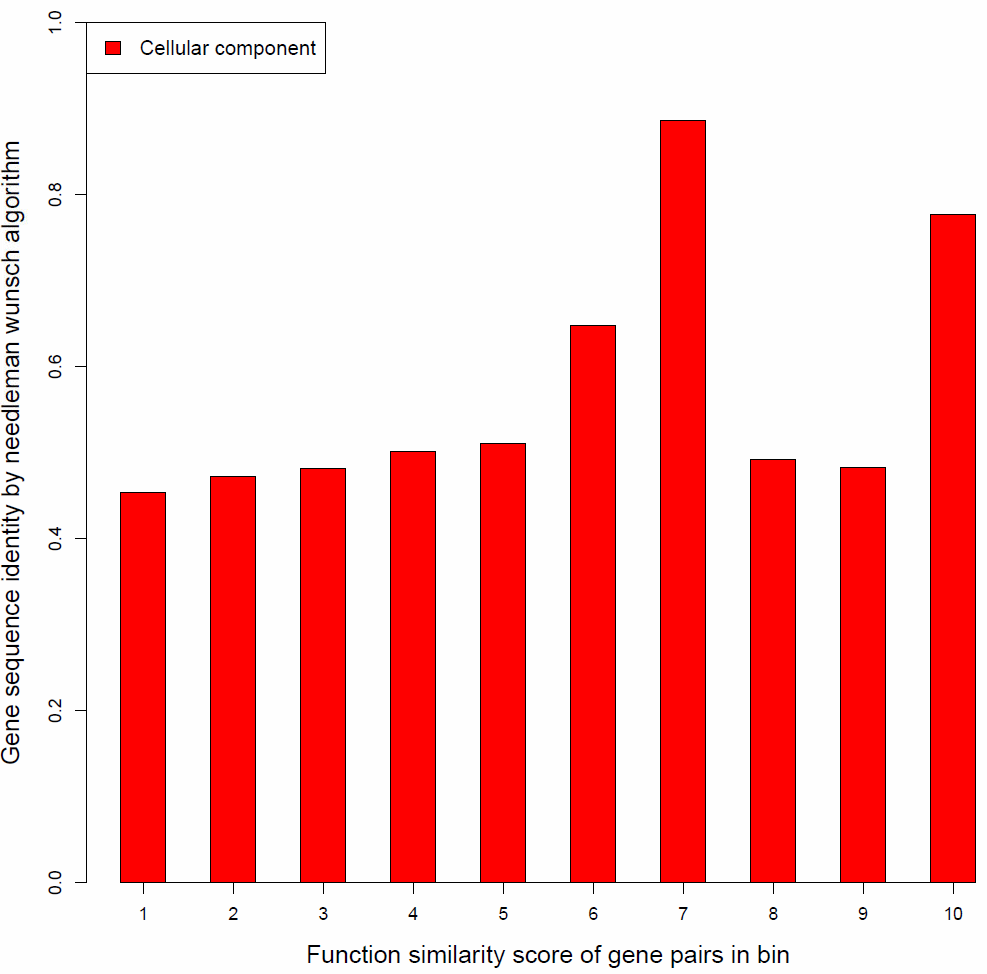

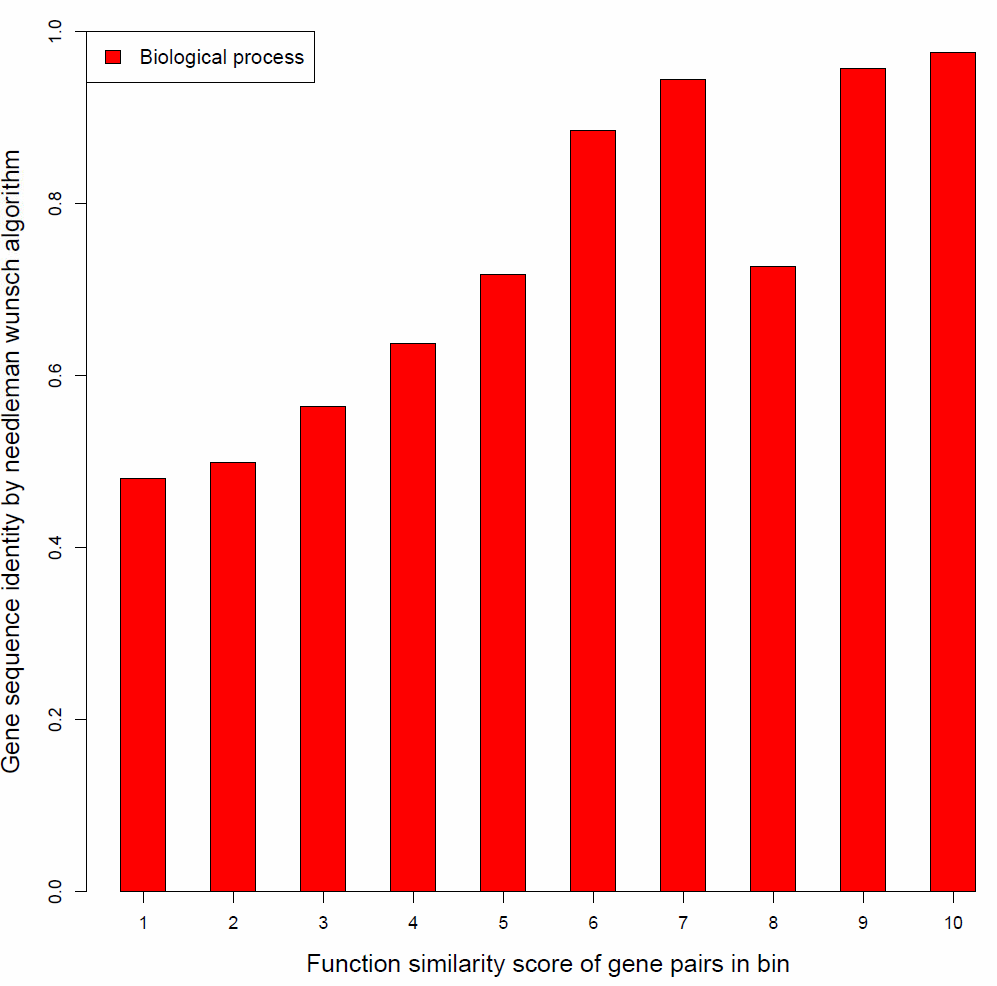


**Figure S7** –The numbers of interacting genes with high function similarity (>0.9) identified by three factors: interaction number, sequence identity, and genomic distance for three function categories (Figure S7(A) – Biological Process, Figure S7(B) – Cellular Component, Figure S7 (C) – Molecular Function). Each sub figure illustrates the number of interacting genes with high function similarity identified by A - interaction number threshold (>= 50 Hi-C reads), B – genomic distance threshold (<=1000000, 2000000, and 1000000 for BP, CC, and MF, respectively), and C – sequence identity (>0.25).

(A) (B) (C)


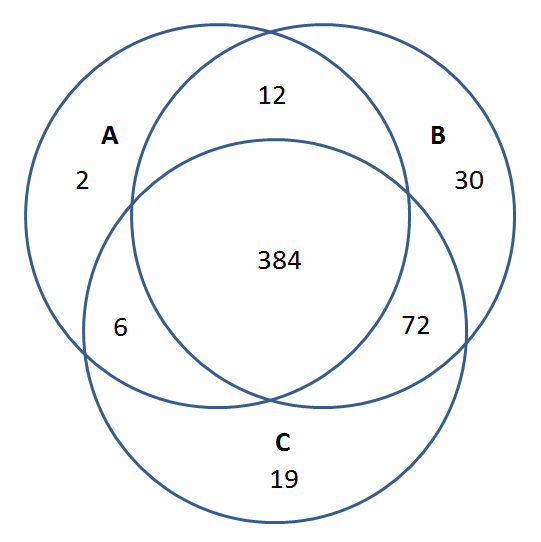

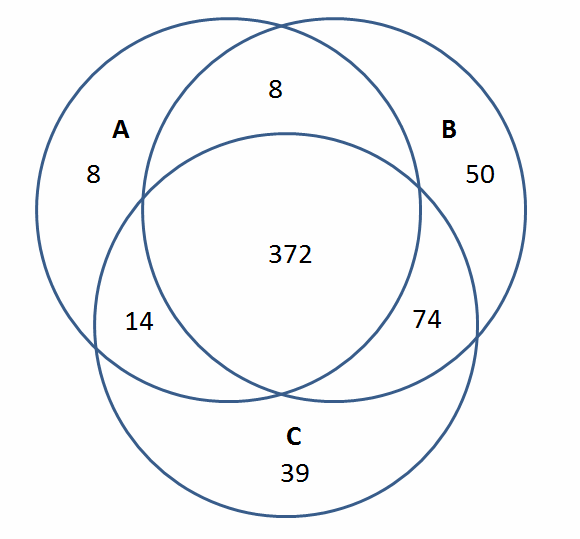

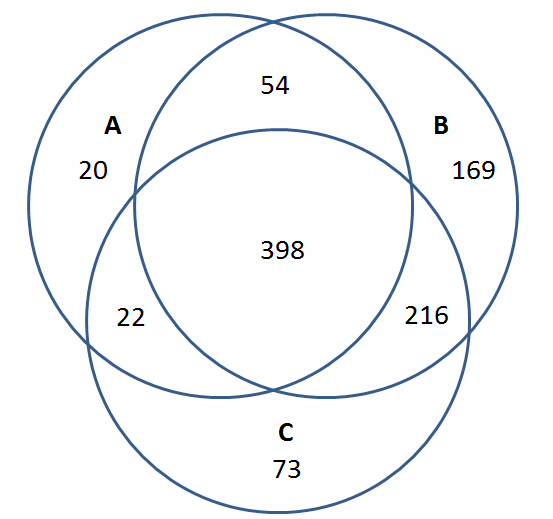


**Figure S8** – Plot of function similarity against sequence identify for substantially interacted gene pairs and non-interacted gene pairs with similar genomic distance for ALL. The correlation between sequence identity and function similarity for ALL is 0.37, 0.25, and 0.43 respectively in three categories.

**Figure S9** – Plot of function similarity against sequence identify for substantially interacted gene pairs and non-interacted gene pairs with similar genomic distance for Call4. The correlation between sequence identity and function similarity for non-interacted gene pairs is 0.34, 0.24, and 0.44 respectively in three categories.

**Figure S10** – Plot of function similarity against sequence identify for substantially interacted gene pairs and non-interacted gene pairs with similar genomic distance for Normal B-cell. The correlation between sequence identity and function similarity for non-interacted gene pairs is 0.29, 0.20, and 0.36 respectively in three categories.

**Figure S11** – Plot of function similarity against sequence identify for substantially interacted gene pairs and non-interacted gene pairs with similar genomic distance for RL. The correlation between sequence identity and function similarity for non-interacted gene pairs is 0.36, 0.25, and 0.43 respectively in three categories.

**Figure S12** – The heat map of gene sequence identity against gene functional similarity score of randomly generated gene-gene networks for three GO function categories (A: Biological Process, B: Cellular Component, C: Molecular Function). Both sequence identity and function similarities are in the range [0, 1]. Deeper color means higher value. The first column list each gene pairs’ sequence identity ranked from high to low, and the second column the corresponding gene function similarity for each gene pair listed in the first column.


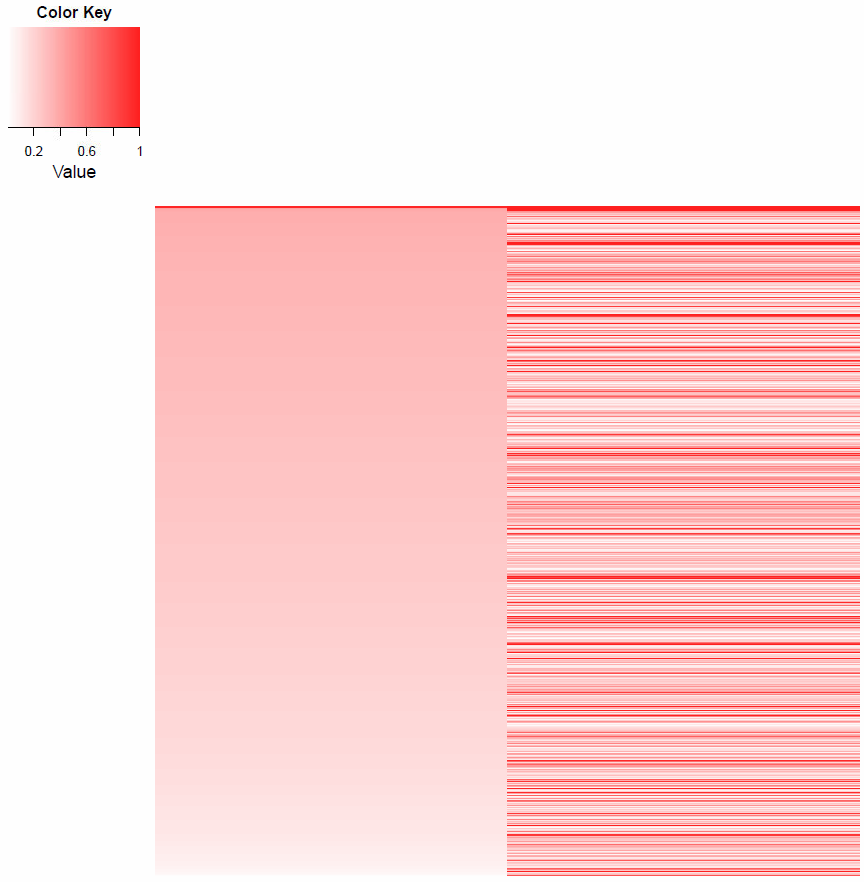
 (A) (B) (C)


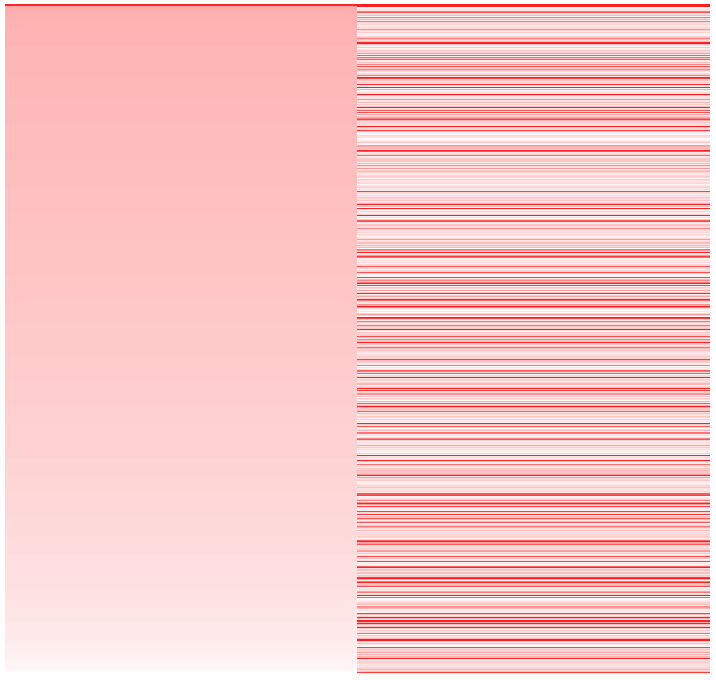

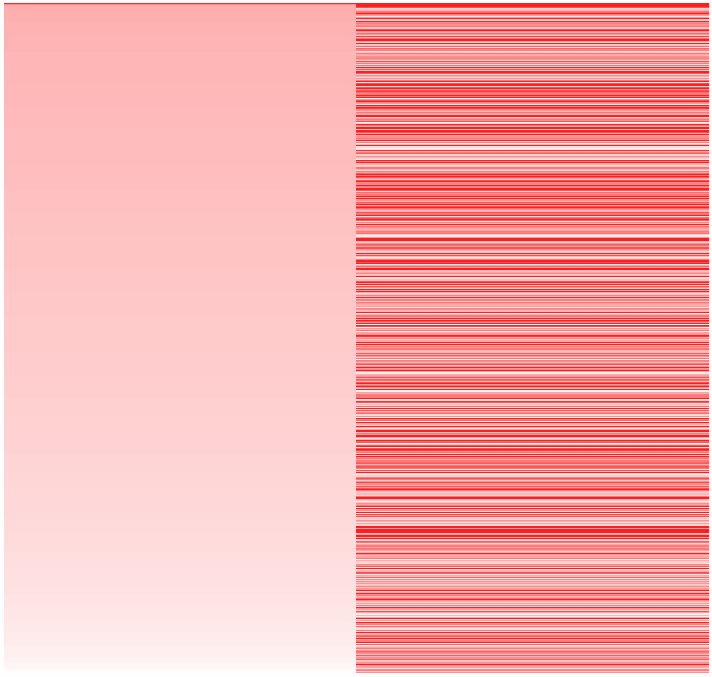

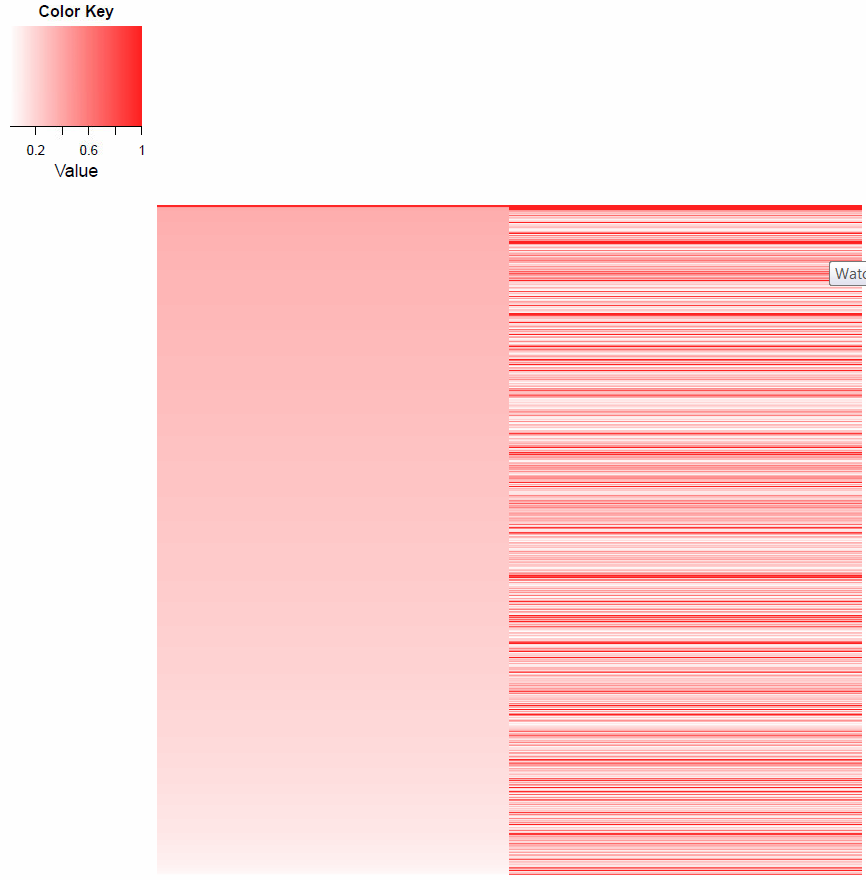

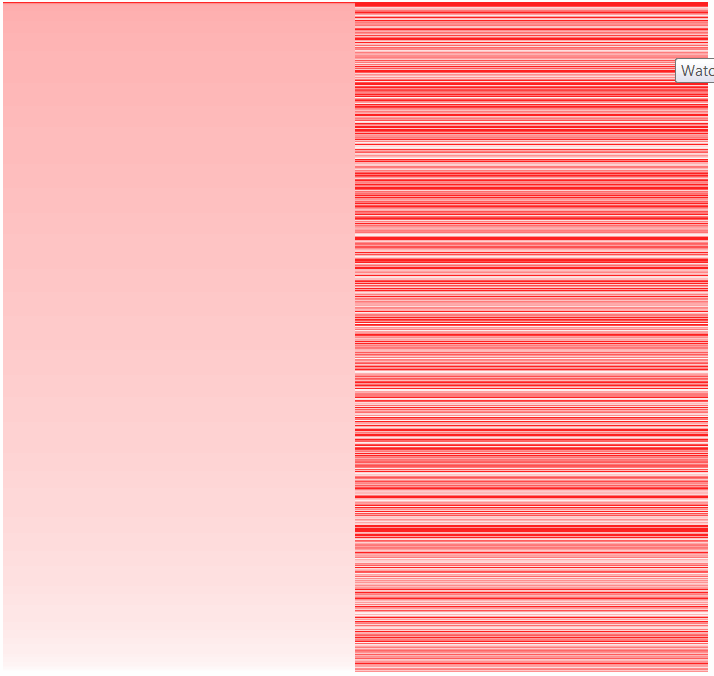

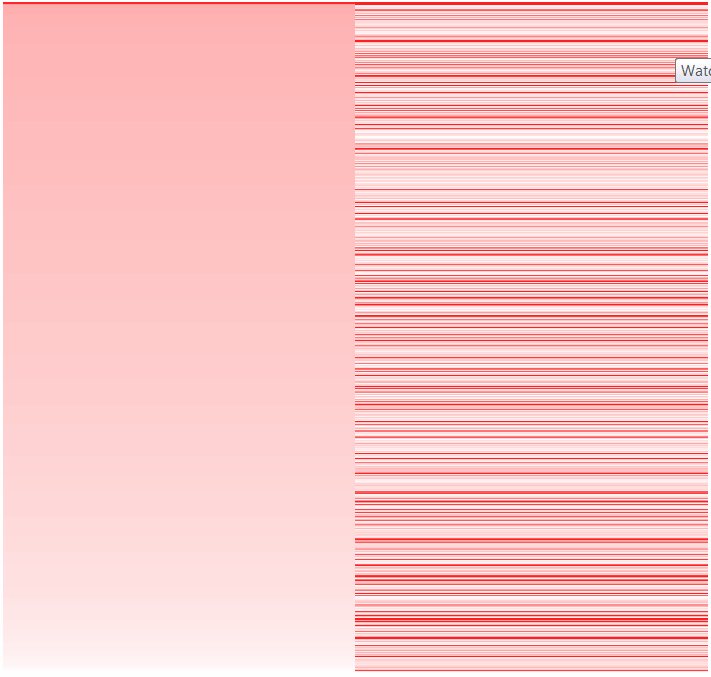


**Figure S13** – The heat map of gene sequence identity against gene functional similarity score of interacting gene pairs on spatial gene-gene networks of the ALL cell at interaction threshold 18 for three GO function categories (A: Biological Process, B: Cellular Component, C: Molecular Function). Both sequence identity and function similarities are in the range [0, 1]. Deeper color means higher value. The first column list each gene pairs’ sequence identity ranked from high to low, and the second column the corresponding gene function similarity for each gene pair listed in the first column.


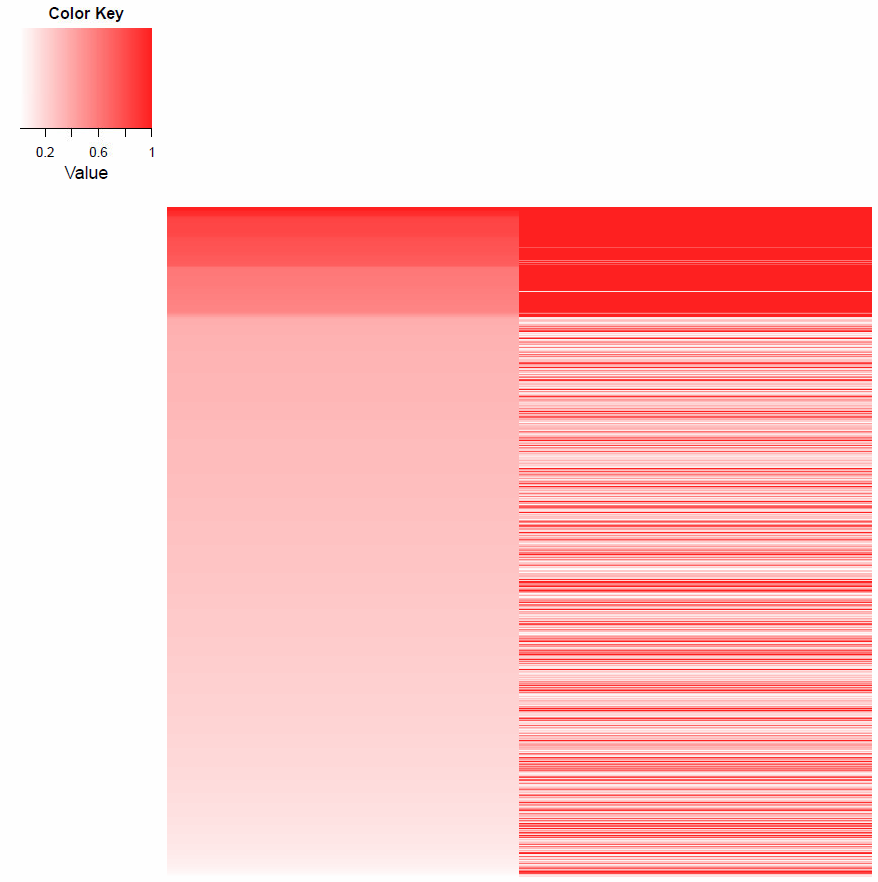
 A B C


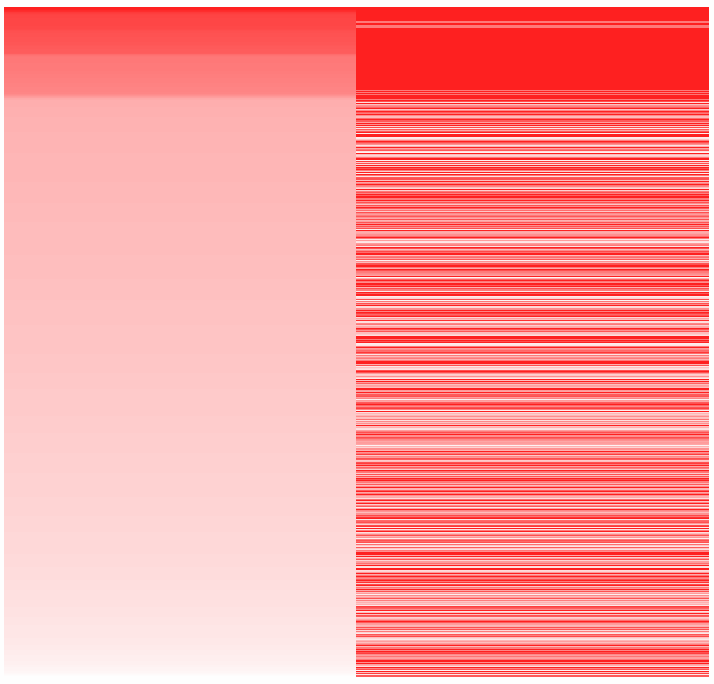

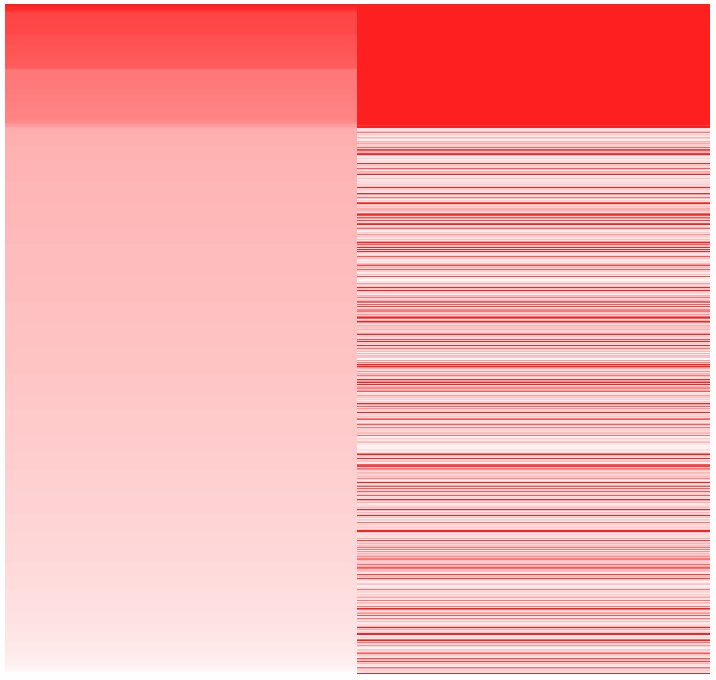

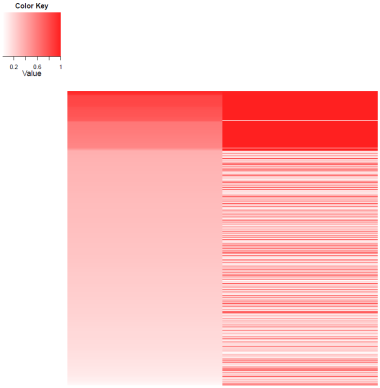

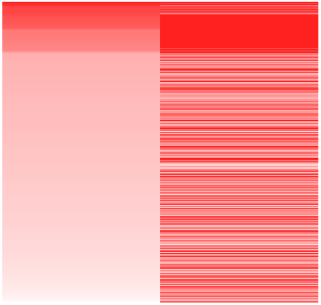

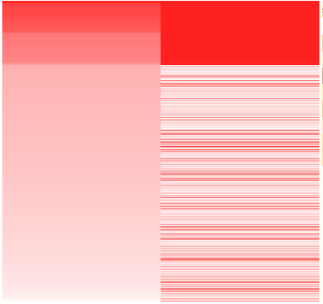


**Figure S14** – The number of interactions against the sequence identity for Call4 cell line, normal B-cell, and RL cell line. Figure S10(A), (B), (C) shows the number of interactions of gene pairs against the sequence identity of Call4 cell line, normal B-cell, and RL cell line respectively. X-axis is the sequence identity, and y-axis is the number of interactions.

(A) (B) (C)


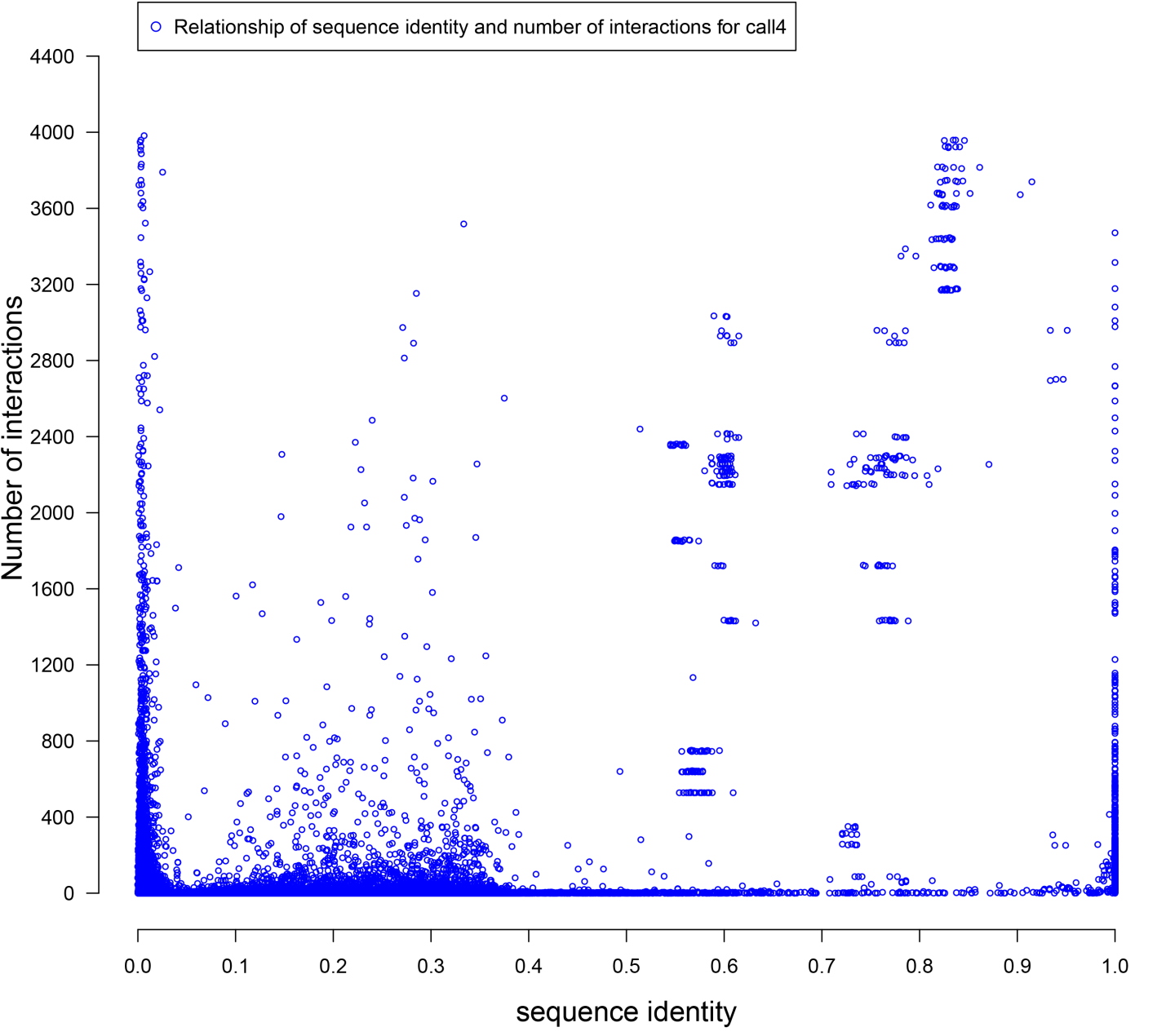

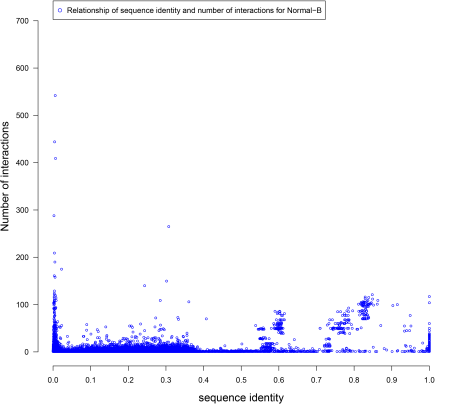

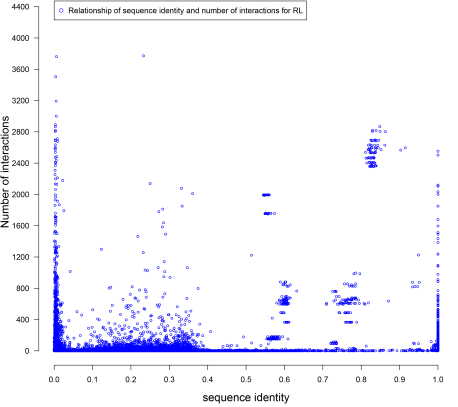


**Figure S15** – The 3D plot of genomic distance, number of interactions and the function similarity in three function categories for gene pairs with relatively long and short genomic distance. Figure S15A, S15B, and S15C show the 3D plot of gene pairs with relatively long genomic distance (longer than the median genomic distance). Figure S15D, S15E, and S15F show the 3D plot of gene pairs with relatively short genomic distance (shorter than the median genomic distance).


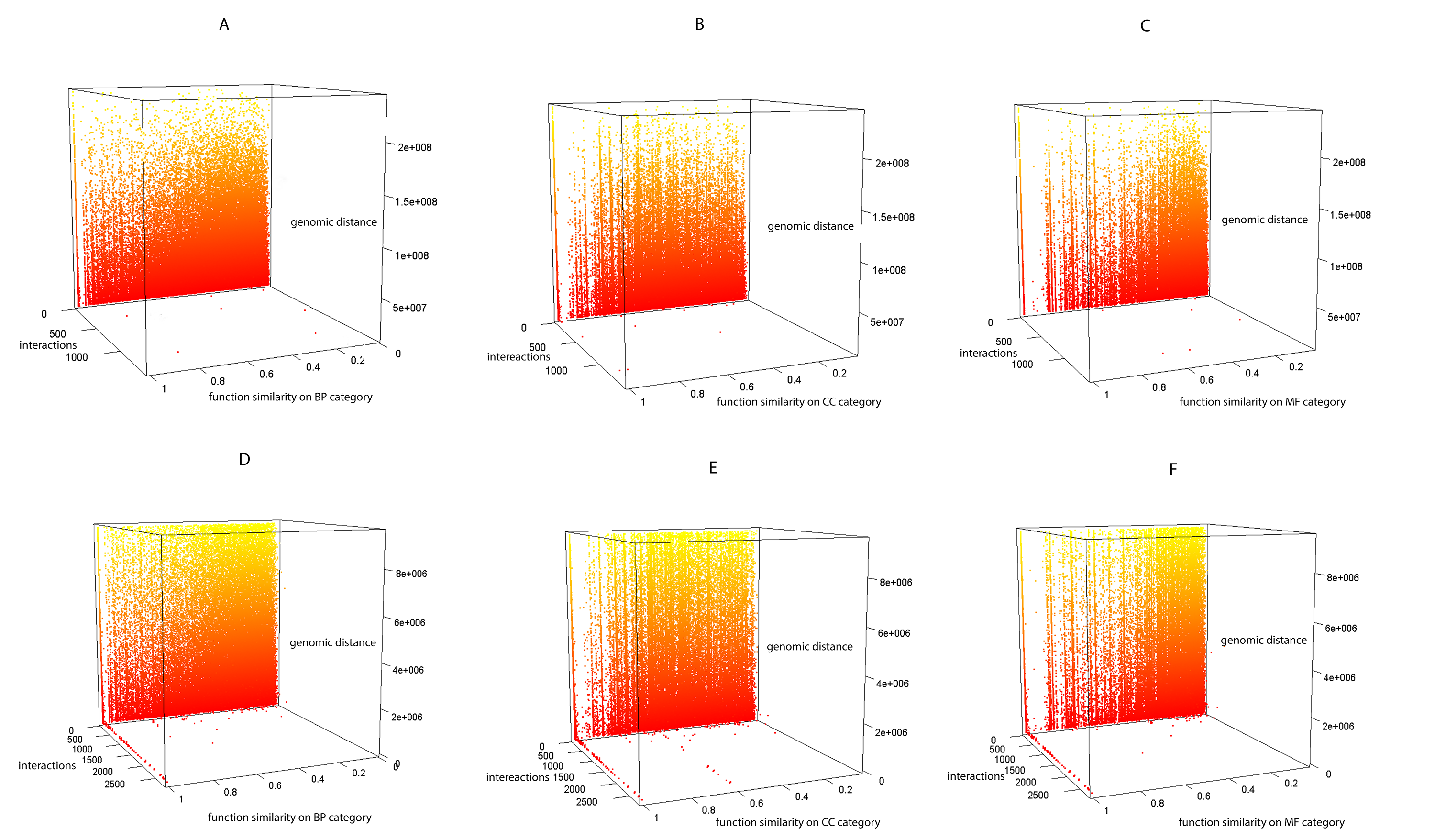


**Table S1** – The twenty genes in the largest cluster of all four cell lines. Interaction threshold 204, 157, 179, and 12 is used for Call4, RL, ALL, and Normal-B cell respectively.

| Call4 | RL | ALL | Normal-B |
| --- | --- | --- | --- |
| GeneID:26025 | GeneID:26025 | GeneID:26025 | GeneID:26025 |
| GeneID:5098 | GeneID:5098 | GeneID:5098 | GeneID:5098 |
| GeneID:56097 | GeneID:56099 | GeneID:56099 | GeneID:56099 |
| GeneID:56098 | GeneID:56100 | GeneID:56100 | GeneID:56100 |
| GeneID:56099 | GeneID:56101 | GeneID:56101 | GeneID:56101 |
| GeneID:56100 | GeneID:56102 | GeneID:56102 | GeneID:56102 |
| GeneID:56101 | GeneID:56103 | GeneID:56103 | GeneID:56103 |
| GeneID:56102 | GeneID:56104 | GeneID:56104 | GeneID:56104 |
| GeneID:56103 | GeneID:56105 | GeneID:56105 | GeneID:56105 |
| GeneID:56104 | GeneID:56106 | GeneID:56106 | GeneID:56106 |
| GeneID:56105 | GeneID:56107 | GeneID:56107 | GeneID:56107 |
| GeneID:56106 | GeneID:56108 | GeneID:56108 | GeneID:56108 |
| GeneID:56107 | GeneID:56109 | GeneID:56109 | GeneID:56109 |
| GeneID:56108 | GeneID:56110 | GeneID:56110 | GeneID:56110 |
| GeneID:56109 | GeneID:56111 | GeneID:56111 | GeneID:56111 |
| GeneID:56110 | GeneID:56112 | GeneID:56112 | GeneID:56112 |
| GeneID:56111 | GeneID:56113 | GeneID:56113 | GeneID:56113 |
| GeneID:56112 | GeneID:56114 | GeneID:56114 | GeneID:56114 |
| GeneID:56113 | GeneID:8641 | GeneID:8641 | GeneID:8641 |
| GeneID:56114 | GeneID:9708 | GeneID:9708 | GeneID:9708 |
| GeneID:8641 |  |  |  |
| GeneID:9708 |  |  |  |
